# Xenobiotica-metabolizing enzymes in the skin of rat, mouse, pig, guinea pig, man, and in human skin models

**DOI:** 10.1007/s00204-018-2232-x

**Published:** 2018-06-18

**Authors:** F. Oesch, E. Fabian, Robert Landsiedel

**Affiliations:** 1 Institute of Toxicology, Johannes Gutenberg-University, Obere Zahlbacherstr. 67, 55131 Mainz, Germany; 2 Experimental Toxicology and Ecology, GV/TB, Z470, BASF SE, Carl-Bosch-Str. 38, 67056 Ludwigshafen, Germany

**Keywords:** Cutaneous xenobiotic metabolism, Species differences, Human skin models, Rat, Mouse, Pig, Guinea pig

## Abstract

Studies on the metabolic fate of medical drugs, skin care products, cosmetics and other chemicals intentionally or accidently applied to the human skin have become increasingly important in order to ascertain pharmacological effectiveness and to avoid toxicities. The use of freshly excised human skin for experimental investigations meets with ethical and practical limitations. Hence information on xenobiotic-metabolizing enzymes (XME) in the experimental systems available for pertinent studies compared with native human skin has become crucial. This review collects available information of which—taken with great caution because of the still very limited data—the most salient points are: in the skin of all animal species and skin-derived in vitro systems considered in this review cytochrome P450 (CYP)-dependent monooxygenase activities (largely responsible for initiating xenobiotica metabolism in the organ which provides most of the xenobiotica metabolism of the mammalian organism, the liver) are very low to undetectable. Quite likely other oxidative enzymes [e.g. flavin monooxygenase, COX (cooxidation by prostaglandin synthase)] will turn out to be much more important for the oxidative xenobiotic metabolism in the skin. Moreover, conjugating enzyme activities such as glutathione transferases and glucuronosyltransferases are much higher than the oxidative CYP activities. Since these conjugating enzymes are predominantly detoxifying, the skin appears to be predominantly protected against CYP-generated reactive metabolites. The following recommendations for the use of experimental animal species or human skin in vitro models may tentatively be derived from the information available to date: for dermal absorption and for skin irritation esterase activity is of special importance which in pig skin, some human cell lines and reconstructed skin models appears reasonably close to native human skin. With respect to genotoxicity and sensitization reactive-metabolite-reducing XME in primary human keratinocytes and several reconstructed human skin models appear reasonably close to human skin. For a more detailed delineation and discussion of the severe limitations see the Conclusions section in the end of this review.

## Introduction

Information on xenobiotic metabolism in the skin becomes more and more important, especially since the ban of the use of animals for safety studies on cosmetics in animals. In the past we published reviews on xenobiotica-metabolizing enzymes (XME) in the skin of man, rat, and pig (Oesch et al. 2007) and on xenobiotica-metabolizing enzymes in the skin of rat, mouse, pig, guinea pig, man and in human skin models (Oesch et al. 2014).

In the mean time important discoveries in the field of xenobiotic metabolism in the skin have been made. Four salient examples:

- The first description of cutaneous aldehyde oxidase activity (Manevski et al. 2014). Missing information on this enzyme had led to several failures of drugs under development (RO1, FK3453, SGX523, zoniporide, BIBX1382, carbazeran: Zhang et al. 2011; Akabane et al. 2011; Diamond et al. 2010; Dalvie et al. 2010; Dittrich et al. 2002; Kaye et al. 1984).
- Sharma et al. (2013a, b) showed that rat and human skin sulfotransferase (SULT) is responsible for biotransformation of the primary nevirapine (Viramune) metabolite 12-hydroxynevirapine to the corresponding reactive benzylic sulfate and that this, in turn, leads to covalent binding to proteins (Sharma et al. 2013a) and to the severe immune-mediated skin rash (Sharma et al. 2013b) caused in rats and in humans as a serious side effect of the HIV combatting drug nevirapine. None of these effects is seen in the mouse skin (no benzylic sulfate, no covalent binding, and no rash). Thus, for this metabolism-dependent immunotoxicity, the rat and not the mouse appear to be a suitable model for human. Similar species differences between generation versus non-generation of reactive benzylic sulfates and consequent toxic effects may occur with other xenobiotics as well.
- The most recent report by Bacqueville et al. (2017) on their three-dimensional model ORS-RHE is to the best of our knowledge the first report on the presence of sulfotransferase (SULT) activity in a three-dimensional model for human skin. As pointed out above this activity is toxicologically especially important for all those xenobiotic compounds which can be metabolized to reactive benzylic sulfates.
- Quite in contrast to the repeatedly reported failure to detect *N*-acetyltransferase (NAT) protein in native human skin, recently Manwaring et al. (2015) reported N-acetylation as the major pathway of metabolism in human skin ex vivo for several aromatic amine hair dyes (percentage of acetylated metabolite 86% for 4-amino-2-hydroxytoluene, 72% for 4-amino-*m*-cresol, 85% for 2-amino-5-ethylphenol, 90% for toluene-2,5-diamine). Recently Manevski et al. (2015) reported for human skin N-acetylation for the first time for procainamide as substrate. Of totally 11 substrates for 5 xenobiotic conjugating enzyme activities the highest observed activity was N-acetylation of para-toluidine. The contrasting results between failure of NAT protein detection and highest observed activity may be related to the notorious lability (and/or polymorphism) of the skin-resident NAT1.

Thus, the purpose of the present review is to update the available information in this rapidly developing and crucially important field. To allow the reader to obtain without going back to our previous reviews (Oesch et al. 2007, 2014) a reasonably complete picture of the available information on the topic of this review we also include in the present review those data already presented in these previous reviews which we estimate to be crucial. In those cases, where we decided not to include the data already presented in the previous reviews we state this in all those places in the text and refer the reader to the previous review (Oesch et al. 2014). It is anticipated that the presentation and update of the collected information will substantially aid in selecting models for general screening and also for individual compounds or problems with sufficient problem-related closeness of their xenobiotic metabolism to in vivo human skin for allowing reasonable predictions of metabolism-dependent toxicities or desired effects of xenobiotic compounds on the human skin.

While many xenobiotic compounds which come into contact with the human skin undergo extensive cutaneous metabolism (as will be detailed in this review) some important consumer products as well as some topical therapeutic agents such as coumarin have been reported to be extensively absorbed through the human skin without being metabolized (Beckley-Kartey et al. 1997; Yourick and Bronaugh 1997). Likewise, Yourick and Bronaugh (1997) as well as Beckley-Kartey et al. (1997) noticed no cutaneous metabolism of coumarin in the rat and Bronaugh et al. (1989) reported lack of cutaneous metabolism for caffeine and DDT. In the minipig skin an important consequence of the virtual lack of cutaneous metabolism of rivastigmin is its high systemic availability upon application to the skin, since this avoids its large first-pass hepatic metabolism (Tse and Laplanche 1998).

Some limitations of the comparability of the data collected in this review have to be taken into account:

- Many cutaneous xenobiotica-metabolizing enzyme activities are close to their limit of quantitation (LOQ) or even to their limit of detection (LOD). This renders accurate determinations difficult. A notable example of this is many cytochrome P450 (CYP)-dependent activities.
- In many (actually in most) cases in the original literature crucial details are not given. Because of the lack of indication whether linearity of the reaction with respect to time and amount of protein were determined or whether the measured activity was just divided by the minutes and by the milligrams used in the experiment, the transformation of the reported activities into unified comparable units would be misleading. The units given in the present review are, therefore, those given in the original literature.
- Very often neither the LOD nor the LOQ are indicated in the original literature. It is, therefore, difficult to judge whether low activities in one instance are actually different from no activities in other instances.
- The different methodologies employed by different research teams add to the difficulties of simple straightforward comparisons of results.

### Preliminary remarks

The numbers presented in the tables of this review attempt to give a crude comparative picture. These numbers refer to different magnitudes and parameters as given in the footnotes to the individual values in the tables. In addition it is imperative to realize that the experimental conditions between the individual investigations reported in the literature of course largely differed. Thus, the numbers are presented to signify that

- the enzymatic activities were present and measurable;
- the activities were high, moderate or low;
- the original authors considered their results as sufficiently trustworthy to present a number.

+ signifies that skin possesses the respective enzymatic activity, but numerical values were either not presented in the original literature or appear uncertain, especially with respect to “per what”? (e.g., per mg of what? Protein? DNA? Total preparation used? Organ?)

## Xenobiotica-metabolizing enzymes in the rat skin

### Cytochromes P450 (CYP)

#### CYP transcript expression

Lee et al. (2001) reported that mRNA of the the following CYPs were expressed in the skin of the rat (Sprague Dawley): 1A1, 1A2, 2B1/2, 2C11, 2E1, 3A1, and 4A1.

#### CYP protein expression

Khan et al. (1989) and Pendlington et al. (1994) observed CYP1A1/A2 and CYP2B1/B2 protein in the rat epidermal preparations, the former noticed in addition CYP1A1 protein after treatment with 3-methylcholanthrene (3-MC), the latter even without pretreatment. The latter study showed that these CYPs also were present in the sebaceous glands.

Zhu et al. (2002) reported the presence of the following CYPs in rat skin microsomes: CYP2B, CYP2C13, CYP2D1, CYP2D4, CYP2E1, CYP3A1, and CYP3A2, their levels being much lower than in the liver (except CYP2D4, which was not observed in the liver). CYP1A1, 1A2, 2C12 were not observed in the skin (method: immunoblotting with mono-specific antibodies).

A novel CYP, CYP2B12, was observed in the preputial gland, but not in the liver (Friedberg et al. 1990, 1992). This CYP turned out to be an arachidonic acid 8,9- and 11,12-epoxygenase present exclusively in sebocytes (Keeney et al. 1998).

The temporal development of CYPs in rat keratinocyte monolayer cultures (serum-free) appears to provide temporal limitations of usefulness for predictions of metabolism-dependent toxicities: CYPs 3A1 and 3A2 proteins were very low, CYP2E1 below detection on day 0, while near confluency of the keratinocytes between days 10 and 14, their levels became similar to those in native skin (Zhu et al. 2002).

CYP reductase (NADPH CYP oxidoreductase) protein (required for CYP activity) has been shown to be present in the rat skin, predominantly in the epidermis (Takahara et al. 1993).

#### CYP catalytic activities

See also Table 1.

**Table 1:** Representative Cytochrome P450 (CYP) basal activities in skin microsomes of various mammalian species

Activity (preferential for)	Human	Rat	Mouse	Guinea pig	Pig
AHH (CYP1 family)	0.24 to 1.35^a^	1.25 ± 0.11^a^	m: 3.3–46^a^; f: 17–21^a^	2.51 ± 0.35^a^	
EROD (CYP1 family)	bd to 35^a^	m: 3.6 ± 0.3; f: 1.5 ± 0.2^a^	m: bd; f: 3–19^a^		4.62 ± 0.54^b^
ECOD (CYP1A,1B,2B,2D6,3A4)	bd to 12^a^	0.36–2.15^a^	10.4–80^a^	3.8 ± 2.7^a^	(13.2 ± 2.5^b^)
MROD (CYP1A2)	bd to +				
PROD (CYP2B)	bd to bq	m: 3.7 ± 1.3; f: 1.8 ± 0.1^a^	m: bq; f: 0.1–1.7^a^	bq	bd
BROD (CYP3A, 2B)		m: 4.4 ± 0.9; f: 2.1 ± 0.2			
Aminopyrine *N*-demethylase (CYP2B, 3A)		1000–4200^a^	+		
Tolbutamide 4-hydroxylation (CYP2C9)	0.46 ± 0.05^b^	0.47 ± 0.04^b^	bd		1.66 ± 0.49^b^
Bufuralol 1-hydroxylation (CYP2D6)	bd	1.33 ± 0.17^b^	9.23 ± 0.67^b^		0.26 ± 0.03^b^
Chlorzoxazone 6-hydroxylation (CYP2E1)	2.83 ± 0.34^b^	bd	20.8 ± 0.5^b^		bd
Para-nitrophenol hydroxylation (CYP2E1)	bd/+	bd^c^	f: 40 ± 10^a^		
Midazolam 1-hydroxylation (CYP3A)	2.35 ± 0.23^b^	0.58 ± 0.09^b^	8.70 ± 0.28^b^		2.32 ± 0.21^b^
Benzoquinoline O-dealkylation (CYP3A)	bd to 76 ± 41^a^				
Erythromycin N-demethylation (CYP3A)	+	bd–270^a^	f: 540–1100^a^		
Testosterone (CYP2A1, 3A4, 19A1, 2C11, 2C19)^d^					+
BP 7,8-dihydrodiol (CYP1A/1B, 2S1)	+	+	+		

More examples and references in the text

*AHH* aryl hydrocarbon hydroxylase, phenolic benzo[*a*]pyrene metabolites determined with 3-hydroxybenzo[*a*]pyrene as standard, *bd* below detection, *BP* benz[*a*]pyrene, *BROD* 7-benzyloxyresofufin *O*-debenzylase, *bq* below quantification, *ECOD* 7-ethoxycoumarin-*O*-deethylase, *EROD* 7-ethoxyresorufin-*O*-deethylase, *f* female, *m* male, *MROD* 7-methoxyresorufin-*O*-demethylase, *PROD* pentoxyresorufin *O*-depentylase

^a^pmol/mg protein/min

^b^pmol/mg protein/h, numbers in brackets: in medium of short-term culture

^c^In epidermal microsomes

^d^Indication of the authors (Jacques et al. 2014) that all these CYPs are present in their pig ear skin model

In early studies Mukhtar and Bickers (1981) reported “aryl hydrocarbon hydroxylase” (AHH) (formation of hydroxylated metabolites from benzo[*a*]pyrene [BP]) activity in skin microsomes of untreated, BP-treated and Aroclor1254-treated neonatal rats (2, 21 and 27% of whole body AHH, respectively). Cutaneous aminopyrene demethylase activity also was observed in the same study. Pham et al. (1989) reported cutaneous dealkylase activity for 7-ethoxyresorufin (EROD) (CYP1-selective), 7-pentoxyresorufin (PROD) (CYP2B-selective), and 7-benzyloxyresorufin (BROD) (broader spectrum for rat CYPs including CYP1A1, 2B1/2 and 3A1). No lauric acid hydroxylation (CYP4A1-selective) was observed.

In more recent studies the following activities (pmol metabolites/h/mg protein) were reported for rat skin microsomes: EROD (CYP1-selective): 1.01 ± 0.14, PROD (CYP2B-selective): below detection at an limit of quantification (LOQ) of 1.87 pmol, tolbutamide 4-hydroxylation (CYP2C9-selective) 0.47 ± 0.04, bufuralol 1-hydroxylation (CYP2D6-selective) 1.33 ± 0.17, chlorzoxazone 6-hydroxylation (CYP2E1-selective) below detection at an LOQ of 12.8 pmol, midazolam 1-hydroxylation (CYP3A-selective) 0.58 ± 0.09 (Rolsted et al. 2008).

*Localization* Early studies already tried to differentiate whether the rat skin CYP activities were present in different skin layers. Bickers et al. (1985) reported metabolism of 2-aminoanthracene to mutagens in 9000 g supernatant (S9) derived from epidermis as well as dermis. Mukhtar et al. (1987) reported that 6-beta-, 7-alpha- and 16-alpha-hydroxylation of testosterone was catalyzed by epidermal (high activity) and by dermal (lower activity) rat skin microsomes. The following observations indicated that CYPs were the responsible catalysts

Requirement of NADPH, the obligatory cofactor for CYP-dependent monooxygenase activity as well as inhibition by the CYP inhibitors SKF-525A and metyrapone.

*Induction* Pioneering work by Wattenberg and Leong (1962) demonstrated increased AHH activity in the rat skin after application of 3-MC to the skin. Subsequent investigations showed increased cutaneous AHH and EROD activities after treatment of the rat skin with BP or with Aroclor 1254 (Mukhtar and Bickers 1981), increased AHH, 7-ethoxycoumarin-*O*-deethylase (ECOD; CYP1A/2B-selective), and EROD activities after topical treatment with 3-MC (Khan et al. 1989) or with 1-nitropyrene or 3-nitrofluoranthene (Asokan et al. 1986), increased para-nitrophenol hydroxylation (CYP2E1-selective) after treatment of the rat skin with clotrimazole (Merk et al. 1989), increased erythromycin demethylase (CYP3A-selective) after irradiation with UVB (Goerz et al. 1996).

Experiments with submerged cultures of skin cells from newborn rats showed that six- to sevenfold increases in AHH and in CYP protein after in vitro exposure to benz[*a*]anthracene. Constitutive AHH activities in these cultures were low in low Ca^2+^ (8 × 10^−5^ M) culture medium, but high when cultured in the presence of high Ca^2+^ concentration (2 × 10^−3^ M). This suggested to the authors that constitutive AHH activity depends on the differentiation status of the skin cells and that this can be modulated by calcium concentration in the culture medium (Guo et al. 1990).

*Inhibition* Moloney et al. (1982a) showed that metyrapone, 5,6-benzoflavone and 7,8-benzoflavone, all of them classical inhibitors of hepatic CYP, also inhibit cutaneous EROD. Mukhtar et al. (1984) showed in microsomal preparations that the antifungal clotrimazole is an exceptionally potent inhibitor of rat cutaneous AHH (IC_50_ 1.2–2.5 × 10^−7^ M), Agarwal et al. (1991) showed that nordihydroguaiaretic acid also is a quite potent inhibitor of cutaneous AHH and of EROD (IC_50_ 4–13 × 10^−5^ M) and Vanden Bossche et al. (1998) showed that the antifungals ketoconazole and miconazole potently inhibit the cutaneous metabolism of retinoic acid (IC_50_ 6.5 × 10^−7^ and 10^−5^ M, respectively), all of this implying the need of caution in the clinical use of these agents.

### Non-CYP oxidoreductases

#### Monoamine oxidase (MAO)

Semak et al. (2004) observed in rat skin MAO activity for serotonin (a preferential MAO A substrate), which was inhibited by pargyline (a selective MAO B inhibitor) (it cannot be concluded from this whether rat skin contains MAO A or MAO B or both).

#### Xanthine oxidase

Under anaerobic conditions 2-nitrofluorene was reduced to the amine by rat skin microsomes in presence of NADPH (low activity) and by rat skin cytosol in presence of 2-hydroxypyrimidine or 4-hydroxypyrimidine or hypoxanthine (high activity). The 2- or 4-hydroxypyrimidine-dependent activity was inhibited by the xanthine oxidase inhibitors oxypurinol and 8-(3-methoxy-4-phenylsulfinylphenyl)-pyrazolo[1,5-*a*]-1,3,5-triazine-4(1*H*)-one (BOF-4272). Partially purified fractions containing xanthine oxidase activity showed immunoreactivity against anti-rat xanthine oxidase. The authors (Ueda et al. 2003) conclude that in rat skin xanthine oxidase is important for the nitroreduction (at least of the substrate used, 2-nitrofluorene), a metabolic step in the activation of nitroarenes to electrophilically reactive mutagens (Vance et al. 1987).

#### Peroxidase

Strohm and Kulkarni (1986) found in rat skin a peroxidase activity for a large array of substrates (benzidine, 3,3′-dimethoxybenzidine, tetramethylbenzidine, *p*-phenylenediamine, hydroquinone, catechol, pyrogallol, para-cresol). The activity was inhibited by cyanide and by azide. Since CYP activities are very low in skin, this peroxidase may provide an important alternative metabolic pathway in the skin.

#### Alcohol dehydrogenase (ADH) and aldehyde dehydrogenase (ALDH)

See also Table 2.

**Table 2:** Representative non-CYP-mediated oxidoreductase activities in skin of various mammalian species

Model substrate (for)	Human	Rat	Mouse	Guinea pig
Benzydamine (FMO)	+			
Methimazole (FMO)			0.35^a^	
Thiobezamide (FMO)			0.32^a^	
Arachidonic acid (COX)	23.5 ± 8.7^b^			
Ethanol^c^ (ADH)	0.3–0.4^d^	2.06^d^	1.1–1.2^d^	0.6^d^
2,6-Dichlorophenolindophenol (NQR)	~ 375^d^		23.4 ± 0.8–159 ± 20^d^	
Menadione (NQR)	7–10^d^	+		
Carbazeran (AO)	1.30^e^			
Zoniporide (AO)	0.164^e^			

More examples and references in the text; only constitutive activities

*ADH* alcohol dehydrogenase, *AO* aldehyde oxidase, *COX* cyclooxygenase, *FMO* flavin-dependent monooxygenase, *NQR* NADH/NADPH quinone reductase

^a^nmol product/mg microsomal protein/min

^b^pg PGE2 formed/mg microsomal protein/min

^c^Beside ethanol ADH activity shown in human skin for 2-butoxyethanol > 2-phenoxyethanol > ethylene glycol > 2-ethoxyethanol as substrates

^d^nmol product/mg cytosolic protein/min

^e^pmol/h/mg skin

*mRNA* Westerlund et al. (2005) found ADH3 mRNA in the epidermis of the rat embryo and adult rat, ADH4 mRNA only in the adult rat, localized in the stratum basale (and partly in the stratum spinosum and granulosum).

*Protein* The following ADH/ALDH proteins were constitutively present in the rat skin: ADH1, ADH3, ALDH1 and ALDH2. They were predominantly localized in the epidermis, hair follicles and sebaceous glands.

*Activity* ADH/ALDH activities were observed in whole and in dermatomed rat skin cytosol. The specific activity (activity per amount of enzyme/per amount of tissue) in the dermatomed skin was twice of that in the whole skin, indicating preferential localization of the activity in the epidermis. The activity for various substrates was 2-butoxyethanol > 2-phenoxyethanol > ethylene glycol > 2-ethoxyethanol > ethanol (Lockley et al. 2005). Activities were increased differentially after topical treatment with ethanol and 2-butoxyethanol in that the activity for the compound of treatment increased predominantly its own metabolism indicating selective induction of enzyme isoforms known to preferentially catalyze the metabolism of short-chain alcohols (ADH1, ALDH1) versus preferentially longer chain metabolizing isoforms (ADH3, ADH4, ALDH1). Dexamethasone increased the activity toward both, ethanol and 2-butoxyethanol, indicating induction of a broader array of isoforms.

#### NAD(P)H:quinone reductase (NQR)

NAD(P)H:quinone reductase [NADH/NADPH quinone oxidoreductase (NQO); DT-diaphorase] is present in rat skin. Expression of its mRNA and its activity is strongly increased by oxidative stress. Its activity is inhibited by the prototypic NQR inhibitor dicoumarol (Rees et al. 1994).

### Hydrolases

See also Table 3.

**Table 3:** Representative xenobiotic hydrolase activities in skin of various mammalian species. Reproduced from Oesch et al. (2014)

Substrate (for)	Human	Rat	Mouse	Pig
Phenyl acetate (E)		1130 ± 25^c^ (micr)		
		3440 ± 1400^d^ (cytos)		
Naphthyl acetate (E)	90 ± 6^a^ (micr)	1500 ± 70^a^ (micr)		28 ± 3^a^ (micr)
	47 ± 3^a^ (cytos)	280 ± 1^a^ (cytos)		85 ± 9^a^ (cytos)
Para-nitrophenyl acetate (E)	91 ± 4^a^ (micr)	2100 ± 100^a^ (micr)		46 ± 6^a^ (minipig micr)
	45–86^a^ (cytos)	380 ± 20^a^ (cytos)		155 ± 18^a^ (minipig cytos)
		188 ± 30^a^ (S9)		
Para-nitrophenyl butyrate (E)		460 ± 127^a^ (S9)		
4-Methylumbelliferone acetate (E)	0.5^a^ (S9)			
Carbaryl (E)		0.2 ± 0.03^c^ (micr)		+
		0.5 ± 0.12^d^ (cytos)		
Fluroxypyr methyl ester (E)		1400^b^ (homogenate)		
Fluroxypyr methylheptyl ester (E)		490^b^ (homogenate)		
Fluazifop-butyl (E)		20 ± 1.5^c^ (micr)		
		400 ± 60^d^ (cytos)		
Methylparaben (E)	~ 400^a^ (micr)			~ 390^a^ (minipig micr)
	~ 550^a^ (cytos)			~ 570^a^ (minipig cytos)
Ethylparaben (E)	~ 420^a^ (micr)			~ 400^a^ (minipig micr)
	~ 520^a^ (cytos)			~ 440^a^ (minipig cytos)
Propylparaben (E)	~ 100^a^ (micr)			~ 150^a^ (minipig micr)
	~ 210^a^ (cytos)			~ 380^a^ (minipg cytos)
Butylparaben (E)	~ 80^a^ (micr)			~ 100^a^ (minipig micr)
	~ 150^a^ (cytos)			~ 140^a^ (minipig cytos)
Benzylparaben (E)	~ 50^a^ (micr)			~ 30^a^ (minipig micr)
	~ 180^a^ (cytos)			~ 170^a^ (minipig cytos)
Ethyl nicotinate (E)		27.3 ± 11.7^a^ (S9)	6.46 ± 1.14^a^ (S9)	
Prodrug esters (E)	+ (many)	+ (many)	+ (many)	+ (alkylazacycloalkan-2-one prodrug esters)
Diethyl hexyl phthalate (E)	+			
Phenanthrene 9,10-oxide (mEH)	2.53^a^ (micr)	0.808^a^ (micr)	1.58^a^ (micr)	
Benz[*a*]anthracene 5,6-oxide (mEH)	0.526^a^ (micr)	0.11^a^ (micr)	0.129^a^ (micr)	
Benzo[*a*]pyrene 4,5-oxide (mEH)	0.175–0.447^a^ (micr)	0.12–0.16^a^ (micr)	0.111–0.172^a^ (micr)	
7-Methylbenz[*a*]anthracene 5,6-oxide (mEH)	0.384^a^ (micr)	0.119^a^ (micr)	0.159^a^ (micr)	
3-Methylcholanthrene 11,12-oxide (mEH)	0.059^a^ (micr)	0.004^a^ (micr)	0.023^a^ (micr)	
Dibenz[*a,h*]anthracene 5,6-oxide (mEH)	0.021^a^ (micr)	0.0015^a^ (micr)	0.003.5^a^ (micr)	
Styrene 7,8-oxide		0.15 ± 0.03^a^ (micr)		
*Cis*-stilbene oxide		0.11–0.0160^a^ (micr)		
*Trans*-stilbene oxide (sEH)		0.027–0.043^a^ (cytos)		

More examples and references in the text; only constitutive activities

*S9* 9000 g supernatant fraction, *cytos* cytosol, *E* esterase, *FPMH* fluroxypyr methylheptyl ester, *mEH* microsomal epoxide hydrolase (EH1; EPH1), *micr* microsomes, *sEH* soluble epoxide hydrolase (EH2; EPH2)

^a^nmol product/min/mg protein

^b^µmol/min/g of tissue

^c^nmol/min/g microsomal fraction

^d^nmol/min/g cytosolic fraction

#### Epoxide hydrolase (EH)

*Microsomal epoxide hydrolase (mEH)* mEH is present in the rat skin, but its specific activity is about 100-fold (Bentley et al. 1976) to 20-fold (Mukhtar and Bickers 1981) lower than in the rat liver. The sequence of its relative activity toward various substrates was the same as in the liver: phenanthrene-9,10-oxide > 7-methylbenz[*a*]anthracene-5,6-oxide ~ benz[*a*] anthracene-5,6-oxide ~ benzo[*a*]pyrene(BP)-4,5-oxide > 3-MC-11,12-oxide > dibenz[*a,h*] anthracene-5,6-oxide (Bentley et al. 1976). Among 26 investigated rat organs mEH specific activity of the skin was among the lowest activities. In the skin mEH activity was mostly localized in the epidermis > dermis > subcutis (Oesch et al. 1977). mEH activity was quantified with BP-4,5-oxide as substrate in these studies after it had been determined that the enzyme responsible for the hydration of this substrate was the same as that for other substrates including the frequently used mEH substrate styrene-7,8-oxide (Oesch and Bentley 1976).

*Cytosolic epoxide hydrolase* Cytosolic EH is present in the rat skin. In preparations containing microsomal and cytosolic cellular components selective substrates may be used for discriminating cytosolic EH from mEH activities: *trans*-stilbene oxide and BP-4,5-oxide for mEH, *cis*-stilbene oxide for cytosolic EH. All these activities were observed in rat skin (Pham et al. 1989). The specific activity of cytosolic EH was about fourfold lower in the skin compared with the liver, the activity in the rat skin was about 1.6-fold lower in the female compared with the male rat.

#### Esterase/amidase

Already early on it had been recognized that rat skin esterases actively hydrolyse many prodrug esters (Sugibayashi et al. 1999; Rittirod et al. 1999; Suppasansatorn et al. 2006) which is of obvious importance for their topical application.

The cutaneous metabolism of the herbicides fluroxypyr methylheptyl ester (FPMH) and fluroxypyr methyl ester (FPM) was extensively investigated. FPM was hydrolyzed considerably faster than FPMH [*V*_max_ 1400 and 490 micromol fluroxypyr (FP) formed/min/g of tissue, respectively]. However, after passage through the rat (and human) skin in vitro, of either of the two compounds only the hydrolysis product, the metabolite FP was present, indicating that systemic exposure was only to the hydrolysis product FP and not to the parent herbicides FPMH or FPM, i.e., indicating cutaneous complete first-pass metabolism (Hewitt et al. 2000a, b). After freezing of the rat skin, the results remained the same, demonstrating considerable robustness of the esterase(s) responsible for this activity.

Prusakiewicz et al. (2006) reported that the hydrolysis efficiency *V*_max_:*K*_m_ for the carboxylesterases substrates para-nitrophenyl acetate and naphthyl acetate in rat skin was lower than in rat liver, but higher than in rat plasma. The efficiency of rat skin is actually unrealistically high if predictions of metabolism-dependent toxicity or desired pharmacological effects are the goal of the investigations: the esterase efficiency in rat skin microsomes was 100- to 1000-fold higher than in human (and pig) skin microsomes, rat skin cytosol was two- to tenfold more efficient than human (and pig) cytosol.

The hydrolysis of Fluazifop-butyl (butyl^®^2-(4-((5-(trifluoromethyl)-2-pyridyl)oxy)phenoxy)propionate) provides a good example for the quantitative differences of the cutaneous esterase activities in the rat skin microsomal versus cytosolic fractions: *V*_max_ was 400 pmol/min/g cytosol versus 20 pmol/min/g microsomes (involvement of carboxylesterase was shown by inhibition experiments using bis-nitrophenol phosphate and paraoxon). Carbaryl and phenylacetate also were hydrolysed by both, microsomal and cytosolic rat skin fractions, the hydrolysis of the latter involving carboxylesterases in the cytosolic and arylesterases in the microsomal fraction (McCracken et al. 1993).

Esterase activity was shown to be predominantly localized in the epidermis and near hair follicles (using fluorescein-5-isothiocyanate diacetate as substrate) (Sugibayashi et al. 1999).

### Conjugating enzymes

#### Glutathione *S*-transferase (GST)

See also Table 4.

**Table 4:** Representative glutathione *S*-transferase (GST) activities in skin of various mammalian species

Substrate (for)	Human	Rat	Mouse	Pig
CDNB (broad spectrum)	20–80^a^ (cytos)	52–247^a^ (cytos)	53.8–1620 ± 140^a^ (cytos)	129 ± 10^a^ (cytos)
	100–290^a,d^			
	40^a,e^			
	15 ± 3^f^			
	0.127^g^			
	ca. 3^a^ (micr)	5.35 ± 0.92^a^ (micr)		
Styrene 7,8-oxide	3.91 ± 0.28^a^ (cytos)	4.80 ± 0.33^a^ (cytos)	5.31 ± 0.33^a^ (cytos)	
*Cis*-stilbene oxide		1.59–1.65^a^ (cytos)		
Benz[*a*]pyrene 4,5-oxide	0.85 ± 0.06^a^ (cytos)	0.90 ± 0.05^a^ (cytos)	1.22 ± 0.09^a^ (cytos)	
3-Methylcholanthrene-11,12-oxide	0.17–0.46^b^ (cytos)		0.62–1.10^b^ (cytos)	
Ethacrynic acid (GST Pi)	5.02 ± 0.41^a^ (cytos)	3.05 ± 0.22^a^ (cytos)	15.5 ± 1.5^a^ (cytos)	
4-Hydroxynonenal (GST A4-4)	ca. 20^a^ (cytos)	< 0.02^c^ (cytos)		
Bromosulphthalein	bd	bd	bd	

More examples and references in the text; only constitutive activities

*bd* below detection, *CDNB* 1-chloro-2,4-dinitrobenzene, *cytos* cytosol, *GST* glutathione *S*-transferase, *micr* microsomes, *PAH* epox polycyclic aromatic hydrocarbon K-region epoxides

^a^nmol product/mg protein/min

^b^nmol product/mg protein/5 min

^c^µg product/mg protein/min

^d^Epidermis (scraped from skin surgical samples)

^e^Dermis

^f^pmol/min/mg protein (sum of 7500 g supernatant + medium)

^g^pmol/h/mg skin (sum of extraction of explant and of medium)

Such as in other organs, glutathione *S*-transferase (GST) specific activity in the rat skin cytosol is considerably higher than in microsomes. For the broad-spectrum substrate 1-chloro-2,4-dinitrobenzene (CDNB) the activity was about tenfold higher in the rat skin cytosolic fraction compared with microsomes (Raza et al. 1991). GST specific activity of the rat skin cytosolic fraction was about sixfold lower than in the rat liver cytosolic fraction (Mukhtar and Bickers 1981), while the cytosolic GSH-specific activity for *cis*-stilbene oxide as substrate was remarkably high in the rat skin, half as high as in the liver (Pham et al. 1989).

GST activities toward the following substrates were observed in the rat skin cytosolic fraction: CDNB, styrene 7,8-oxide, BP 4,5-oxide, ethacrynic acid, leukotriene A4, but not toward cumene hydroperoxide and bromosulfophthalein.

Using CDNB as broad-spectrum substrate rat skin cytosolic GST activity increased from day 5 to day 67 from 48.3 to 65.7 nmol/min/mg protein (Raza et al. 1991).

Rat epidermal GST activity toward CDNB was increased 1.8-fold by topical treatment with clotrimazole (Mukhtar et al. 1984).

Localization of rat skin GST was reported to be almost exclusively in the sebaceous glands (Pendlington et al. 1994).

Glutathione (GSH) levels varied largely from below detection to 73 ± 28 nmol glutathione/mg skin protein (Rees et al. 1995; Adamson et al. 1996; Jewell et al. 2000; Yarat et al. 2001; Korać and Buzadzić 2001; Romeu et al. 2002; Tunali et al. 2004).

Various GST classes were found in the rat skin cytosol: Mu and Pi (predominantly Pi), but not alpha classes (in contrast to the human skin which possesses GST protein of the alpha but not the mu class) (Raza et al. 1991).

GST A4-4, which was below detection in the rat skin cytosolic fraction, was induced by UVB irradiation and then localized in epidermis and sebaceous glands (Hiratsuka et al. 1999). This alpha-class GST is important for the detoxication (conjugation) of the toxic 4-hydroxy-2(*E*)-nonenal (HNE), which arises by lipid peroxidation from from ω-6 polyunsaturated fatty acids.

Ya–Ya and Ya–Yc alpha-class GSTs and the selenium-containing glutathione peroxidase GSH Px (Se-GSH Px) can reduce cholesterol 7α- and 7β-hydroperoxides (7α- and 7β-hydroperoxycholest-5-en-3b-ols). These cholesterol hydroperoxides are toxic and are aging markers in the rat skin (Ozawa et al. 1991). Hiratsuka et al. (1997) reported that in the rat skin Ya GSTs were below detection and Se-GSH Px were present at very low levels which in all likelihood leads to high levels of the toxic cholesterol 7-hydroperoxides. The most abundant GST in the rat skin was Yb2–Yb2 > Yc–Yc > Yp–Yp > Yb1–Yb1 > Yb1–Yb2.

#### UDP-glucuronosyltransferase (UGT)

See also Table 5.

**Table 5:** Representative UDP-glucuronosyl (UGT) and sulfotransferase (SULT) activities in skin of various mammalian species

Substrate (for)	Human	Rat	Mouse	Pig
4-Methylumbelliferone (UGT)	1.3 ± 0.2^a^; 2.89^c^		11.1 ± 9.65^d^	
Bilirubin (UGT)	+	15–23^a^		
4-Hydroxybiphenyl (UGT)		< 0.08^a^		
7-Hydroxycoumarin (UGT)				< 0.333 ± 0.03^d^
1-Naphthol (UGT)		2.5–13^a^		
3-Hydroxybenzo[*a*]pyrene (UGT)		0.08 ± 0.01^a^		
Testosterone (UGT)		< 0.09^a^		
17β-Estradiol 3-glucuronidation	0.054^c^			
17β-Estradiol 17-glucuronidation	0.006^c^			
Indomethacin (UGT)	0.274^c^			
Diclofenac (UGT)	0.055^c^			
Triclosan (UGT)	1.95^c^			
4-Nitrophenol (UGT)			13.7 ± 2.6^h^	
Salicylic acid (UGT)	0.0062–0.016^a^			
Acetaminophen (UGT)		+ (tentative)		+ (tentative)
Acetaminophen (SULT)		+ (tentative)		+ (tentative)
7-Hydroxycoumarin (SULT)				< 0.183 ± 0.029^d^
4-Nitrophenol (SULT)	Traces − 0.18 ± 0.02^e,f^			
Minoxidil (SULT)	0.013^c^			
Minoxidil (SULT1A1)	0.21 ± 0.02^e^	0.006^g^		
Triclosan (SULT)	1.63^c^			
Dopamine (SULT)	0.60 ± 0.05^e^			
17β-Estradiol 3-sulfation	0.180^c^			
12-Hydroxynevirapine	+	+	bd	

More examples and references in the text; only constitutive activities

*UGT* UDP-glucuronosyltransferase, *SULT* sulfotransferase, *bd* below detection

^a^nmol product/min/mg microsomal protein

^b^nmol/min/g of skin

^c^pmol/h/mg skin (sum of extraction of explant and of medium)

^d^nmol/h/g of skin analyzed in the culture medium

^e^nmol/h/mg “high speed supernatant” protein

^f^pmol/min/mg protein (sum of 7500 g supernatant + medium)

^g^nmol product/min/mg cytosolic protein

^h^µmol product/mg microsomal protein/min

Rat skin UGT activity has been shown for several substrates: 1-naphthol, *N*-hydroxy-2-naphthylamine, 3-hydroxy-BP, BP-7,8-dihydrodiol, bilirubin, while activity toward testosterone as substrate was not observed (Bock et al. 1980; Moloney et al. 1982b; Pham et al. 1989). The Km values were determined for 1-naphthol and bilirubin and shown to be much lower than in the rat liver, while the specific activities were 10–50% of those in the liver (Moloney et al. 1982b; Pham et al. 1989). Treatment with 3-MC led to modest increases of the activities in the skin (Moloney et al. 1982b).

Vienneau et al. (1995) showed a protective role of UGT in rat skin fibroblasts against the genotoxicity of BP as well as NNK (4-[methylnitrosamino]-1-[3-pyridyl]-1-butanone) and Kim et al. (1997) showed such a role for phenytoin.

Hardingham and Phelps (1968) reported that the UGT cofactor UDP-glucuronic acid was present at a concentration of 0.08 mM “in skin cellular water” and at a concentration of 28 ± 2 nmol/g wet tissue in the neonatal rat skin (Table 6).

**Table 6:** Representative *N*-acetyltransferase (NAT) activities in skin of various mammalian species

Substrate	Human	Rat	Mouse	Pig
Para-aminobenzoic acid^a^	0.45 ± 0.17^b^	3–6^b^	+	
Para-phenylenediamine	0.41–3.68^b^	+ (tentative)		+ (tentative)
Para-aminophenol	+	+ (tentative)		+ (tentative)
Para-toluidine	0.63–3.03^b^, 4.76^c^			
Procainamide	0.036^c^			
2-Aminofluorene		ca. 1^b^	ca.3^b^	

More examples and references in the text; only constitutive activities

^a^Preferential substrate of the major skin NAT, NAT1

^b^nmol product/mg cytosolic protein/min

^c^pmol/h/mg skin (sum of extraction of explant and of medium)

#### Sulfotransferase (SULT)

Investigations on SULT in the rat skin are scarce.

Wong et al. (1993) noticed that the antihypertonic drug minoxidil, which possesses interesting hair growth promoting activity, appears in the rat skin to be a substrate of SULT1A1. This conclusion was based on the following observations: The SULT activity of the rat skin cytosolic fraction toward minoxidil was thermostable and was inhibited by para-nitrophenol and by 2,6-dichloro-4-nitrophenol [which are good inhibitors of SULT1A1 (Wang et al. 2009)], but not by low concentrations of tyramine or dopamine (substrates of SULT1A3). During this SULT-mediated metabolic transformation of minoxidil ^35^S was transferred from sodium ^35^sulfate to minoxidil indicating that the rat skin is capable of synthesizing PAPS (3′-phosphoadenosine-5′-phosphosulphate), the obligatory cofactor for the SULT activity and that, therefore, the metabolism-requiring hair growth promoting activity of minoxidil could be provided by the skin itself.

Recently Sharma et al. (2013a, b) showed that rat (and human) skin SULT is responsible for biotransformation of the primary nevirapine (Viramune) metabolite 12-hydroxynevirapine to the corresponding reactive benzylic sulfate and that this, in turn, leads to covalent binding to proteins (Sharma et al. 2013a) and to the severe immune-mediated skin rash (Sharma et al. 2013b) caused (in rats and in humans) as a serious side effect of the HIV combatting drug nevirapine. None of these effects is seen in the mouse skin (no benzylic sulfate, no covalent binding, no rash). Thus, for this metabolism-dependent immunotoxicity the rat and not the mouse appear to be a suitable model for human. Similar species differences between generation versus non-generation of reactive benzylic sulfates and consequent toxic effects may occur with other xenobiotics as well.

#### *N*-Acetyltransferase (NAT)

Semak et al. (2004) showed that in the rat skin serotonin is acetylated to the precursor of melatonin and that this reaction requires the NAT cofactor acetyl coenzyme A. He, furthermore, showed that this reaction was inhibited by the “Cole bisubstrate inhibitor”. He concluded that the rat skin possesses arylamine and arylalkylamine *N*-acetyltransferase activities and that serotonin was a substrate for both of them.

Two NATs are important for xenobiotic metabolism: NAT1 and NAT2 [a third NAT gene, NAT3, has been described by Walraven et al. (2006)]. Dressler and Appelqvist (2006) tentatively identified NAT1 to mediate in the rat skin the acetylation of para-aminophenol. NAT1 activity is not stable leading to quite variable results (Fabian et al. 2013).

## Xenobiotica-metabolizing enzymes in the mouse skin

### Cytochromes P450 (CYP)

#### CYP transcript expression

Du et al. (2009) reported the expression in the CD-1 mouse skin of the following CYPs on the RNA level: Highest expression: *Cyp4f13* and *4f16*, intermediate: *Cyp4f18* and *4f39*, low: *Cyp4f14, 4f17, 4f37*, highly variable (in some animals below detection): *Cyp4f15* and *Cyp4f40*. The expression of additional CYPs (*Cyp1a1, Cyp1b1, Cyp2e1*) was observed in the skin of C57BL/6J mice by Flowers et al. (2011).

In the mouse skin the expression of several *Cyp* genes was demonstrated on the RNA level. Determination of (CD-1) mouse cutaneous RNA levels by real-time polymerase chain reaction showed transcripts arising from *Cyp4f13* and *4f16*(most abundant), *Cyp4f18* and *4f39* (intermediate), *Cyp4f14, 4f17*, and *4f37* (low), and from *Cyp4f15* and *Cyp4f40* (highly variable or too low to measure in some animals) (Du et al. 2009). In addition, the expression of the *Cyp1a1, Cyp1b1, Cyp2e1* genes was observed in (C57BL/6J) mouse skin (Flowers et al. 2011).

Interestingly, recent studies by Borland et al. (2014) showed that the AhR-dependent induction of the expression of several CYPs (as well as several phase II xenobiotic-metabolizing enzymes) in mouse skin and in mouse keratinocytes required the presence of peroxisome proliferator-activated receptor β/δ (PPARβ/δ). They compared Pparβ/δ null mice primary keratinocytes as well as whole animals versus their wild types (and also Pparβ/δ-knock down with wild-type human HaCat keratinocytes) which showed an intriguing cross talk between PPARβ/δ and AhR signaling and consequent inductions of CYP mRNA expression (and expression of phase II xenobiotic-metabolizing enzymes).

Recent studies by Elentner et al. (2015) showed that the transcription factor pregnane X receptor (PXR) is expressed in mouse skin and its expression is increased by topical treatment with the skin carcinogen 7,12-dimethylbenz[*a*]anthracene (DMBA). Cyp1a1, Cyp1b1 and Cyp3a11, but not Ahr expression in the skin also were enhanced by DMBA. 48 h after application of DMBA, skin expression of Pxr and Cyp1a1 mRNAs were markedly increased, Cyp1b1 and Cyp3a11 only moderately and Ahr not at all. Pxr mRNA expression increased with time. Expression of Cyp1b1 and Cyp1a1 mRNAs peaked at 4 h, expression of Cyp3a11 mRNA peaked at 24 h (upregulation of PXR in the DMBA-treated skin was confirmed at the protein level).

#### CYP protein expression

The presence of CYP protein in the mouse skin was observed by several researchers: Pohl et al. (1976) and Pendlington et al. (1994) (CYP1A1/A2 and CYP2B1/B2 localized predominantly in the epidermis and sebaceous glands), Jugert et al. (1994) (CYPs 1A1/2, 2B1/2, 2E1, and 3A), Saarikoski et al. (2005b) (CYP2S1), Arora et al. (2013) (presence of CYP and cytochrome b5).

Increases in CYP protein in the mouse skin by pretreatment with the following agents were reported: TCDD (Pohl et al. 1976), β-naphthoflavone (Pendlington et al. 1994), dexamethasone (Jugert et al. 1994), CDNB (Saarikoski et al. 2005b), treatment with a tumor initiation–promotion protocol (one topical application of 500 nmol 7,12-dimethylbenz[*a*]anthracene for 2 weeks, then 1.7 nmol TPA twice per week for 6 weeks) (Arora et al. 2013). The latter increase was markedly diminished by simultaneous treatment with *Azadirachta indica* leaf extract known to be active against skin cancer (and cancer of several other organs) (Koul et al. 2006).

#### CYP catalytic activities

See also Table 1.

*Constitutive activities* Pohl et al. (1976) reported hydroxylation of BP and aniline and deethylation of 7-ethoxycoumarin in mouse skin microsomes, Jugert et al. (1994) AHH, EROD, PROD, para-nitrophenol hydroxylase, and erythromycin *N*-demethylase activities, Das et al. (1985) AHH, and ECOD.

The following activities were reported by Rolsted et al. (2008) for mouse skin microsomal preparations (pmol metabolites/h/mg protein): EROD 95.4 ± 4.2, PROD below detection (LOQ 1.87 pmol), tolbutamide 4-hydroxylation (CYP2C9-selective substrate) below detection, bufuralol 1-hydroxylation (CYP 2D6-selective substrate) 9.23 ± 0.67, chlorzoxazone 6-hydroxylation (CYP2E1-selective substrate) 20.8 ± 0.5, midazolam 1-hydroxylation (CYP3A-selective substrate) 8.70 ± 0.28.

20-Hydroxylation of leukotriene B4 was demonstrated by Du et al. (2009) in mouse skin.

The comparative study by Storm et al. (1990) showed that in mouse skin AHH (3.35 ± 0.07 pmol/min/mg protein) and ECOD (10.4 ± 1.4 pmol/min/mg protein) activities were much higher than in human skin (0.24 ± 0.08 pmol/min/mg protein and not detected, respectively), whereas guinea pig skin had similar activities (2.51 ± 0.35 and 3.8 ± 2.7 pmol/min/mg protein, respectively) as the mouse skin.

##### Modulation of activities

*Increases* of moue skin enzymatic activities were reported for the following agents and the following activities: TCDD: AHH, EROD (Pohl et al. 1976), β-naphthoflavone: ECOD (Pendlington et al. 1994), coal tar: AHH, ECOD (Das et al. 1985), dexamethasone: AHH (Briggs and Briggs 1973), EROD, PEROD, para-nitrophenol hydroxylase, and erythromycin *N*-demethylase (Jugert et al. 1994), retinoic acid: LTB4 hydroxylation (Du et al. 2009).

*Decreases* of moue skin enzymatic activities were reported for the following agents and the following activities: chronic UVB irradiation: AHH, ECOD (Das et al. 1985), tumors formed by UVB irradiation: AHH, ECOD (Das et al. 1985).

Modi et al. (2012) observed that mice lacking Langerhans cells, signatory epidermal dendritic cells, were protected from chemical carcinogenesis by the polycyclic aromatic hydrocarbon (PAH) DMBA, which is known to be metabolized to the proximate mutagen/carcinogen DMBA-3,4-dihydrodiol by CYP1B1 and subsequent action of EH (Buters et al. 2003). Langerhans cells efficiently metabolized DMBA to DMBA 3,4-dihydrodiol. Application of this metabolite, DMBA 3,4-dihydrodiol overcame tumor resistance in mice deficient in Langerhans cells.

Arora et al. (2013) reported constitutive presence of AHH activity in male LACA mice skin (46.3 ± 2.1 pmol 3-hydroxy-BP formed/min/mg microsomal protein) and its modest but significant (*p* ≤ 0.05) increase after treatment with a tumor initiation–promotion protocol (one topical application of 500 nmol 7,12-dimethylbenz[*a*]anthracene for 2 weeks followed by 1.7 nmol TPA twice weekly for 6 weeks). This increase was diminished by a co-treatment with the cancer preventive *A. indica* leaf extract (300 mg/kg body weight orally on days alternate to DMBA/TPA treatment) [this extract is active against several types of cancers including skin cancer (Koul et al. 2006)].

Dwivedi et al. (2013) reported measurable AHH and EROD activities in Swiss Albino female mice skin (0.34 ± 0.03 pmol/min/mg protein and 0.28 ± 0.02 pmol/min/mg protein, respectively; not clear whether this refers to S9 or total homogenate protein) which was reduced by topical treatment with commercial benzanthrone (150 nmol/mouse twice weekly for 30 weeks), the reduction of which was (partially or totally, respectively) prevented by simultaneous topical treatment with ascorbic acid (amount not given).

Basketter et al. (2008) concluded that CYP1A1 converts eugenol to a sensitizing metabolite based on their observation that in contrast to wild-type mice 1A1-knockout mice are not sensitized by eugenol.

*Localization* Coomes et al. (1983) separated mouse skin cells by metrizamide and Percoll gradient centrifugations. ECOD activities were highest in the fractions containing a high percentage of sebaceous cells. In a similar study Reiners et al. (1992) reported that ECOD and EROD activities were lowest in basal keratinocytes and increased with increasing differentiation of the keratinocytes. Topical treatment with dibenz[*a,c*]anthracene increased EROD activities mostly in the undifferentiated keratinocytes (1850-fold!), but also in the more highly differentiated keratinocytes, albeit much less.

*Differentiation*/*age-dependence* In the fetal mouse skin *Cyp2b19* expression coincided in space and time of onset with the expression of loricrin and, therefore, represents a marker of late differentiation (Keeney et al. 1998). After birth CYP2B19 is expressed in the differentiated keratinocytes of the epidermis, sebaceous glands, and hair follicles selectively in granular cells (which represent differentiated keratinocytes). In cultures CYP2B19 is upregulated in parallel with the upregulation of calcium-induced differentiation and with the upregulation of *Loricrin* and *Profilaggrin* (late differentiation genes) (Keeney et al. 1998).

Keeney et al. (1998) reported that arachidonic acid is a substrate of recombinant CYP2B19, generating among other arachidonic acid metabolites 11,12- and 14,15-epoxyeicosatrienoic acids in proportions similar to those in the mouse epidermis suggesting CYP2B19 to be the major producer of these biologically active EETs in the mouse epidermis.

Mouse skin EROD, ECOD and NADPH CYP reductase activities (but not aldrin epoxidase activity) became markedly lower in senescent mice (Williams and Woodhouse 1996).

### Non-CYP oxidoreductases

See also Table 2.

#### Cyclooxygenase (Cox) (prostaglandin synthase)

Sivarajah et al. (1981) observed that in the presence of arachidonic acid BP-tetrols (7/8,9,10-tetrol and 7,10/8,9-tetrol) were formed from *trans*-7,8-dihydroxy-7,8-dihydro-BP by mouse skin microsomes. The reaction was inhibited by indomethacin. The authors concluded that the reaction was catalyzed by Cox.

Cox (Cox2) mRNA expression was increased upon UVB irradiation (600 J/cm^2^) in wild-type but not in Ah receptor knockout mice. This shows that Cox2 expression is controlled by Ah receptor and that this receptor is activated by UVB irradiation (Fritsche et al. 2007).

#### Alcohol dehydrogenase (ADH) and aldehyde dehydrogenase (ALDH)

Cheung et al. (2003b) showed that ADH1, ADH3, ALDH1, ALDH2 and ALDH3 protein was present in mouse (BALB/c and CBA/ca) skin. However, ADH2 was not observed in the mouse skin although it was observed in the mouse liver (and in the human skin and liver).

Localization of these ADH and ALDH enzymes in the skin of the mouse was predominantly in the epidermis, sebaceous glands and hair follicles.

Apparent *V*_max_ of 1.07–1.21 nmol/min/mg protein were determined for the mouse cutaneous ADH activities with ethanol as substrate, considerably higher than in the human skin (0.32–0.41). 1 mM of the ADH inhibitor 4-methyl pyrazole led to a decrease of the activity with ethanol as substrate by 60–70% (Cheung et al. 2003b).

#### NAD(P)H:quinone reductase (NQR)

Remarkably, NQR (also called NADH/NADPH quinone oxidoreductase NQO; DT-diaphorase) specific activity in the mouse skin is almost as high as in mouse liver (about 80% of the hepatic activity) (compare AHH activity: 100-fold lower in the mouse skin compared with the mouse liver) (Merk et al. 1991).

After percoll gradient separation of mouse skin cells into populations differing in their differentiation stages the specific NQR activities turned out to be considerably lower in the most differentiated populations. Topical treatment of mouse skin led to an about threefold increase in the NQR specific activity subsequently measured in these separated populations. The increase was similar in all populations (Reiners et al. 1992).

Arora et al. (2013) reported constitutive presence of NQR (DT-diaphorase) activity in male LACA mice skin (0.03 ± 0.003 µmol DCPIP reduced/min/mg 10 000 g supernatant protein) which was significantly (*p* ≤ 0.05) decreased after treatment with a tumor initiation–promotion protocol (one topical application of 500 nmol 7,12-dimethylbenz[*a*]anthracene for 2 weeks followed by 1.7 nmol TPA twice weekly for 6 weeks). This decrease was reversed by a co-treatment with the cancer preventive *A. indica* leaf extract (300 mg/kg body weight orally on days alternate to DMBA/TPA treatment) [this extract is active against several types of cancers including skin cancer (Koul et al. 2006)].

Dwivedi et al. (2013) reported measurable NQR activities in Swiss Albino female mice skin (23.4 ± 0.8 nmol/min/mg S9 protein) which was increased by topical treatment with commercial benzanthrone (150 nmol/mouse twice weekly for 30 weeks), the increase of which was partially prevented by simultaneous topical treatment with ascorbic acid (amount not given).

### Hydrolases

See also Table 3.

#### Epoxide hydrolase (EH)

Low activity of microsomal EH was observed in the mouse (SKH hairless) skin (about 20-fold lower than in the liver of the same mouse strain). Neither chronic UVB irradiation nor tumorigenesis significantly changed the specific activity (Das et al. 1985).

EH3 (ABHD9), a first member of a new epoxide hydrolase family with high activity for fatty acid epoxides, was observed by Decker et al. (2012) in the mouse skin. Among several mouse organs, the expression of EH3 (ABHD9) mRNA was highest in lung, skin, and upper gastrointestinal tract.

### Conjugating enzymes

#### Glutathione *S*-transferase (GST)

See also Table 4.

Mouse skin possesses Mu and Pi (predominantly Pi), but not alpha classes of GST proteins (in contrast to the human skin which possesses GST protein of the alpha but not the mu class) (Raza et al. 1991). Their localization was predominantly in sebaceous glands. They expressed enzymatic activities toward the following substrates: CDNB, ethacrynic acid, BP 4,5-oxide, styrene 7,8 oxide, and leukotriene A4. Cumene hydroperoxide and bromosulfophthalein did not measurably serve as substrates.

Mouse skin (MFl/hr hairless) GST activity toward the broad-spectrum substrate CDNB was increased about fourfold after pretreatment with phenobarbital. This GST activity was localized predominantly in the epidermis and sebaceous glands (Pendlington et al. 1994).

After percoll gradient separation of mouse skin cells the highest activity toward the broad-spectrum substrate CDNB was observed in the fraction containing the highest percentage of sebaceous cells (Coomes et al. 1983).

GST activities toward CDNB as substrate in mouse (SKH hairless) skin were about twofold increased by chronic UVB irradiation and reduced back to control activities in the skin tumors caused by this irradiation (Das et al. 1985).

Arora et al. (2013) reported constitutive presence of GST activity toward CDNB as substrate (1.62 ± 0.14 µmol/min/mg cytosolic protein) in male LACA mice which was not significantly changed after treatment with a tumor initiation–promotion protocol (one topical application of 500 nmol 7,12-dimethylbenz[*a*]anthracene for 2 weeks followed by 1.7 nmol TPA twice weekly for 6 weeks) nor by a treatment or co-treatment with the cancer preventive *A. indica* leaf extract (300 mg/kg body weight orally on days alternate to DMBA/TPA treatment) [this extract is active against several types of cancers including skin cancer (Koul et al. 2006)].

Dwivedi et al. (2013) reported measurable GST activities with CDNB as substrate in Swiss Albino female mice skin (140 ± 34 nmol/min/mg S9 protein).

In the mouse skin leukotriene A4 methyl ester was conjugated with GSH to the leukotriene C4 methyl ester (Agarwal et al. 1992). Leukotriene C4 is further metabolized to the leukotrienes D4 and E4 which cause allergic up to anaphylactic reactions in the skin.

Skin GST activity for the broad-spectrum substrate CDNB was lower in the mouse compared with pig < rat < human (Jewell et al. 2000).

#### UDP-glucuronosyltransferase (UGT)

After percoll gradient separation of mouse (hairless HRS/J) skin cells UGT activities toward the broad-spectrum substrate 4-methylumbelliferone were highest in the fraction containing the highest percentage of sebaceous cells (Coomes et al. 1983).

Arora et al. (2013) reported constitutive presence of UGT activity toward para-nitrophenol as substrate in male LACA mice skin (13.7 ± 2.6 µmol glucuronide formed/min/mg microsomal protein) which was not significantly changed after treatment with a tumor initiation–promotion protocol (one topical application of 500 nmol 7,12-dimethylbenz[*a*]anthracene for 2 weeks followed by 1.7 nmol TPA twice weekly for 6 weeks) nor by a treatment or co-treatment with the cancer preventive *A. indica* leaf extract (300 mg/kg body weight orally on days alternate to DMBA/TPA treatment) [this extract is active against several types of cancers including skin cancer (Koul et al. 2006)].

#### *N*-Acetyltransferase (NAT)

Not much is known about NAT in mouse skin. Stanley et al. (1997) reported that they observed by immunohistochemistry in sebaceous glands and cells in the shafts of the hair follicles NAT2, the mouse homologue of the skin-resident human NAT1 (Kawamura et al. 2008).

## Xenobiotica-metabolizing enzymes in the guinea pig skin

### Cytochromes P450 (CYP)

See also Table 1.

Storm et al. (1990) reported that guinea pig skin possesses AHH and ECOD activities which were much higher than in human skin [but similar to (Sencar) mouse skin].

In cultured guinea pig epidermal cells AHH activity increased with increasing proliferation and returned to initial levels around days 5–7 of culture. The AHH activity also increased upon treatment of the cells with benzanthracene. The benzanthracene-dependent rise in activity remained at a constant ratio also at days 5–7 (Thiele et al. 1987).

Guinea pig skin microsomes converted leukotriene B4 (LTB4) to 20-hydroxy-LTB4. This leukotriene B4 omega-hydroxylase activity depended on the presence of oxygen and NADPH, was inhibited by SKF-525A and by CO allowing for the conclusion that the reaction was catalyzed by CYP (Mukhtar et al. 1989).

Young et al. (1997) reported that guinea pig skin possesses CYP reductase localized predominantly in the sebaceous glands and to a lower degree in hair follicle cells and in the epidermis.

### Non-CYP oxidoreductases

#### Lipoxygenase

Ruzicka and Printz (1982) showed that guinea pig skin is very active in the metabolism of arachidonic acid, predominantly by the lipoxygenase pathway leading to hydroxyeicosatetraenoic acids (HETE). Lipoxygenase activity was predominantly localized in the epidermis and to a much lower degree in the dermis.

#### Cyclooxygenase (COX)

Ruzicka and Printz (1982) reported that the minor pathway of arachidonic acid metabolism in guinea pig skin, the cyclooxygenase (Cox) pathway, converted arachidonic acid to prostaglandin D2 and to a lesser amount to prostaglandin E2. The Cox activity was localized predominantly in the epidermis and to a lower degree in the dermis.

#### Alcohol dehydrogenase (ADH) and aldehyde dehydrogenase (ALDH)

Guinea pig (Dunkin-Hartley) skin was shown by Cheung et al. (2003b) to possess ADH1, ADH3, ALDH1 and ALDH2, but not ADH2, protein, although ADH2 protein was clearly seen in the guinea pig liver (and in human skin and liver). In addition, ALDH3 was not observed in the guinea pig skin (but in the human, rat and mouse skin). Localization of ADH and ALDH was mainly in the epidermis, sebaceous glands and hair follicles.

ADH activity toward ethanol as substrate was somewhat higher in the guinea pig skin (apparent *V*_max_ 0.6 ± 0.8 [!] nmol/min/mg protein) compared with the human skin (0.32–0.41 nmol/min/mg protein). 1 mM of the ADH inhibitor 4-methyl pyrazole reduced the guinea pig skin ADH activity by about 80% (Cheung et al. 2003b).

### Hydrolases

#### Esterase/amidase

As far as reported, esterase/amidase activities were minimal in guinea pig skin. Morris et al. (2009) showed that ethyl, propyl and butyl *O*-acyl prodrug esters of haloperidol were hydrolysed very slowly, and the longer chain octyl and decyl *O*-acyl prodrug esters of haloperidol still much slower during permeation across freshly excised full-thickness guinea pig skin. In addition, a skin extract showed very low hydrolysis rates toward these prodrug esters (after 50 h only 9.56, 6.58, 9.17, 6.07, 3.56% haloperidol were liberated from ethyl, propyl, butyl, octyl and decyl haloperidol *O*-acyl esters, respectively).

### Conjugating enzymes

#### Glutathione *S*-transferase (GST)

Guinea pig epidermis and dermis possess a very high activity of GSH-dependent prostaglandin H2/D2 isomerase converting prostaglandin H2 to prostaglandin D2. This isomerization is catalyzed by GST. Prostaglandin D2 was practically the only product formed from prostaglandin H2 by guinea pig skin homogenate (Ruzicka and Printz 1982).

## Xenobiotica-metabolizing enzymes in the pig skin

### Cytochromes P450 (CYP)

See also Table 1.

Chang et al. (1994) observed that isolated viable pig skin converts parathione to paraoxone, a reaction presumed to be catalyzed by CYP.

Rolsted et al. (2008) reported that minipig skin microsomes catalyzed the following CYP prototypic biotransformation reactions (expressed in pmol metabolites/h/mg protein): EROD 4.62 ± 0.54, PROD below detection (LOQ 1.87 pmol), tolbutamide 4-hydroxylation 1.66 ± 0.49, bufuralol 1-hydroxylation 0.26 ± 0.03, chlorzoxazone 6-hydroxylation below detection (LOQ 12.8 pmol), midazolam 1-hydroxylation 2.32 ± 0.21.

In the medium of pig ear skin explant cultures Jacques et al. (2010a) observed as a direct or indirect result of CYP activity the following metabolites of 7-ethoxycoumarin: 1.3 ± 1.2 pmol/h/mg protein of 7-hydroxycoumarin, 7.71 ± 0.73 pmol/h/mg protein of 7-hydroxycoumarin glucuronide, and 4.24 ± 0.66 pmol/h/mg protein of 7-hydroxycoumarin sulfate.

Pig ear skin microsomes and pig ear skin explant cultures metabolized BP to hydroxylated BPs, as well as to their glucuronides and sulfates and to BP-diols, BP-catechols and BP-diones (Jacques et al. 2010b).

Recently Jacques et al. (2014) showed that in pig ear skin explant cultures testosterone was metabolized to the derivatives hydroxylated at positions 7α, 6β, 16α and at several not identified additional positions. Further metabolites were androstenedione, hydroxy-androstenediones, 16α-∆4-androstenedione, androsterone and epiandrosterone. The major metabolite was a hydroxy-androstenedione (position of the hydroxyl group not identified) which was formed at 3.09 ± 0.33 nmol/h/mg (presumably mg tissue). From the presence of these metabolites the authors hypothesize that CYP2A1, 3A4, 19A1, 2C11 and 2C19 are present and functional in their pig ear skin model. Incubation of microsomes derived from from the skin of pig ears yielded (in presence but not in absence of an NADPH generating system) 16α-hydroxytestosterone, androstenedione and epiandrosterone, but none of the other metabolites observed in the culture medium.

### Hydrolases

#### Epoxide hydrolase (EH)

Pig skin microsomes and pig ear skin explant cultures converted BP to BP-diols and diol-glucuronides (Jacques et al. 2010b).

#### Esterase/amidase

See also Table 3.

Chang et al. (1994) showed that isolated pig skin hydrolysed parathion and carbaryl to para-nitrophenol and naphthol. Rangarajan and Zatz (2001) showed that micro-Yucatan pig skin hydrolyzed alpha-tocopheryl acetate to free alpha-tocopherol, whereby stratum corneum did not possess this hydrolytic activity. Bonina et al. (2003) showed that pig skin activated six 1-alkylazacycloalkan-2-one prodrug esters of ketoprofen to free ketoprofen.

The involvement of carboxylesterases was shown for the following reactions catalyzed by pig skin: The hydrolysis of methyl-, ethyl-, butyl- and benzyl parabens was inhibited by the carboxylesterase inhibitors bis-nitrophenylphosphate and by paraoxon (Jewell et al. 2007). Prusakiewicz et al. (2006) showed that the prototypical carboxylesterases substrates para-nitrophenyl acetate and naphthyl acetate were hydrolysed by pig skin microsomes with similar efficiencies (*V*_max_/*K*_m_) (1.2–4.2/min/mg) as was seen with human skin (1.3–4.2/min/mg). Pig skin cytosol also hydrolysed these substrates with relatively similar efficiencies (18–61/min/mg) as seen with human skin cytosol (2.4–67/min/mg).

On the other hand, Bi and Singh (2000) showed that the hydrolysis of [Arg8]-vasopressin was catalyzed in the pig skin (at least predominantly) by aminopeptidase as shown by the bestatin-mediated inhibition.

Oh et al. (2002) reported an interesting influence of the formulation on the fate of methyl paraben. It was efficiently hydrolysed to 4-hydroxybenzoic acid by pig (Yucatan micropig) skin. However, transesterification leading to ethyl para-hydroxybenzoate was the predominant reaction as soon as ethanol was present.

### Conjugating enzymes

#### Glutathione *S*-transferase (GST)

Pig skin possesses GST activity for the broad-spectrum substrate CDNB, higher than the mouse skin, but lower than rat and human skin (see Table 4). The GST cofactor GSH was present in pig skin at 18.6 ± 1.5 nmol GSH/cm^2^ (Jewell et al. 2000).

#### UDP-glucuronosyltransferase (UGT)

van de Sandt et al. (1993) showed that from 2-isopropoxyphenol [metabolite of 2-isopropoxyphenol (Propoxur)] in pig skin glucuronides were formed in amounts equal to sulfates (in contrast to human skin which formed only sulfate).

7-Hydroxycoumarin (metabolite of 7-ethoxycoumarin) was in pig skin culture transformed to its glucuronide as the major metabolite (7.71 ± 0.73 pmol/h/mg protein in culture medium) (Jacques et al. 2010a).

Benz[*a*]pyrene was metabolized in pig ear skin explant cultures and by pig skin microsomes to glucuronides of the hydroxybenzo[*a*]pyrenes as the major metabolites and also to glucuronides of the benzo[*a*]pyrene diols as minor metabolites (Jacques et al. 2010b).

Zalko et al. (2011) found that the major metabolite of bis-phenol A in pig skin culture was its mono-glucuronide.

#### Sulfotransferase (SULT)

van de Sandt et al. (1993) reported that pig skin converted 2-isopropoxyphenol metabolite of 2-isopropoxyphenyl *N*-methylcarbamate (Propoxur) to the sulfate and glucuronide in approximately equal amounts (human skin converted 2-isopropoxyphenol only to the sulfate).

7-Hydroxycoumarin (metabolite of 7-ethoxycoumarin) was converted by pig ear skin explant cultures to the sulfate as the second most abundant metabolite (14.67 ± 2.29 pmol/h/cm^2^ found in the culture medium) (Jacques et al. 2010a).

Benz[*a*]pyrene was converted by pig skin microsomes and by pig skin explant cultures to the hydroxybenzo[*a*]pyrene sulfates as second most abundant metabolites (Jacques et al. 2010b).

Zalko et al. (2011) found that by pig ear skin fresh explant cultures, but not after freezing, bis-phenol A was converted to its mono-sulfate (second most abundant metabolite).

#### *N*-Acetyltransferase (NAT)

Dressler and Appelqvist (2006) showed that upon topical application para-phenylenediamine is metabolized to *N,N*′-diacetyl-para-phenylenediamine, para-aminophenol to paracetamol (para-acetylaminophenol) as only metabolites in blood plasma, with no parent compounds left indicating that systemic exposure after topical application of the parent compounds is exclusively to these metabolites.

## Xenobiotica-metabolizing enzymes in the human skin including skin-derived cells and cell lines as well as reconstructed skin models

Wiegand et al. (2014) systematically compared human skin xenobiotic metabolism in respect to basal and induced phase I and phase II enzymes regarding gene/protein expression as well as enzyme activity in four in vitro test systems (2-dimensional and 3-dimensional) with that in native human skin. They concluded that reconstructed skin models are a valuable tool for safety assessment with regard to xenobiotic metabolism, but that the 3D models mirrored the in vivo situation more realistically than did the monolayer cultures. The individual results will be discussed in the following under the corresponding enzyme activities.

Recently Sowada et al. (2014) reported that bacterial isolates from human skin (all 21 isolates investigated) degraded benzo[*a*]pyrene as sole source of carbon and energy demonstrating that not only enzymatic transformations by the skin itself, but from skin-resident bacteria as well represents an important source for metabolic activations and inactivations of xenobiotica. The human skin which spans an area of about 1.8 m^2^ harbors more than 200 bacterial genera with up to 10 million bacterial cells/cm^2^ (the core microbiome on human skin often being composed of Actinobacteria, Firmicutes, Bacteroidetes and Proteobacteria, these phyla containing thousands of species, some of which having large metabolic versatility) (Mathieu et al. 2013; Tralau et al. 2015). It, therefore, should be kept in mind that the microbiome on the skin and its potentially quite divergent abundance and composition in vivo versus in vitro may substantially contribute to differences of results of in vivo experiments versus in vitro models, possibly even versus ex vivo experiments.

### Cytochromes P450 (CYP)

Xenobiotica-metabolizing CYPs activities (and proteins) are very low in untreated human skin. In several instances low activities (and protein) were reported by some investigators, but not detected by others. On the other hand, the expression of xenobiotica-metabolizing CYPs RNAs was clearly demonstrated by several investigators. However, the low or non existent levels of xenobiotica-metabolizing CYPs proteins and activities in many cases are dramatically inducible by several xenobiotic inducers.

#### CYP transcript expression

*Human skin* Yengi et al. (2003) reported the presence of the following CYPs mRNA in full-thickness human skin biopsies: CYP1B1, CYP2B6 (not present in some individuals), CYP2D6, CYP3A4; low expression: CYP1A1, CYP2C9, CYP2C18, CYP2C19, CYP2E1 and CYP3A5 mRNAs; not detected: CYP1A2, 2A6, and 2C8.

In human whole skin CYP mRNA expression was demonstrated for totally 36 CYPs samples by Baron et al. (2008), including 1A1, 1A2, 1B1, 2A6/7, 2B6/7, 2C9, 2C18, 2C19, 2D6, 2E1, 2S1, 3A4/7, 3A5.

Smith et al. (2006) reported that in adult human full-thickness skin biopsies the following CYPs mRNA were increased by treatment with coal tar: CYP1A1, CYP1A2, CYP1B1, CYP2C18; by treatment with all-*trans*-retinoic acid: CYP26 and NADPH P450 reductase (CYP1A1 and CYP1A2 RNAs decreased); by treatment with clobetasol 17-propionate: CYP3A5.

Janmohamed et al. (2001) reported that in adult human skin the CYP2B6 RNA, but not CYP2A6 and 3A4 mRNAs decreased with increasing age. Localization of these three CYP mRNAs was in the epidermis, sebaceous glands and hair follicles.

*Cells in culture* (for an overview of the cell lines see Table 7). Janmohamed et al. (2001) observed that the levels of CYP2A6, 2B6, 3A4 RNAs were much higher in the skin as compared with the keratinocyte cultures derived from the respective skin. No CYP2A6 and 3A4 mRNA was seen in the human keratinocyte cell line HCaT, while CYP2B6 mRNA levels were about the same as in skin (Janmohamed et al. 2001). Rea et al. (2002) reported that in the human keratinocytic cell line SIK the mRNs of the following CYPs were present: 4B1, 11A1, 4F3, 2A7, 4F2 and 51.

**Table 7:** Overview of frequently used cell lines for studies on cutaneous xenobiotic-metabolizing enzymes. Reproduced from Oesch et al. (2014)

Cell line	Type	Reference and/or origin
HaCaT	Spontaneously immortalized aneuploid immortal, but non-tumorigenic human keratinocytic cell line	Boukamp et al. (1988)
NCTC 2544	Human breast-skin keratinocyte-derived cell line	Bakken et al. (1961)
KeratinoSens^®^	Derived from human keratinocytes; possesses a luciferase gene under control of the human aldo keto reductase AKR1C2 antioxidant response element (ARE)	Givaudan
LuSens	Derived from human keratinocytes; possesses a luciferase gene under control of the rat NADH/NADPH quinone reductase NQO1 antioxidant response element (ARE)	Bauch et al. (2011) and Ramirez et al. (2014)/BASF SE
U937	Established from a diffuse histiocytic lymphoma displaying many monocytic characteristics	DSMZ, Braunschweig
THP-1	Derived from the blood of a patient with acute monocytic leukemia	Qin (2012)/DSMZ, Braunschweig

*DSMZ* Deutsche Sammlung von Mikroorganismen und Zellkulturen GmbH (German Collection of Microorganisms and Cell Cultures)

In NOK (“normal human oral keratinocytes” in culture) and in SVpgC2a (a Siman virus 40 T antigen-immortalized oral keratinocyte line) Vondracek et al. (2002) observed similar levels of the mRNAs of CYP2B6/7, CYP 2E1, CYP reductase and the aryl hydrocarbon receptor nuclear translocator, while that of CY1A1, 1B1 and aryl hydrocarbon receptor RNA levels were higher in the SVpgC2a cells.

Saeki et al. (2002) found in cultured keratinocytes CYP1A1, 1B1, CYP2C, 2E1, 3A5 and 4B1 mRNAs, in cultured Langerhans cells CYP1A1, 1B1, 1E1, 3A4 and 3A7 mRNAs, in fibroblasts CYP1A1, 1B1, 2A6, 2C, 2D6, 2E1, CYP3A5 and 3A7 mRNAs, in cultured melanocytes CYP1A1, 1B1, 2A6 and 2E1 mRNAs. In contrast to the further above-mentioned studies CYP1A2, 2A7, 2B6 and 3A4 mRNAs were not detected in keratinocytes in this investigation (the keratinocytes were cultured for 36 h only, in presence of 1.5 mM calcium). Du et al. (2006a, b) investigated the expression of CYPs mRNAs in keratinocytes after 6 days in cultures [monitoring the upregulation of transglutaminase 1 and keratin 10 mRNA and the morphology to make sure that the spinous stage (the first differentiation stage) was reached]. They found in these cells expression of the RNAs of the following CYPs: 1A1, 1A2, 1B1, 2C9, 2C18, 2C19, 2D6, 2E1, 2J2, 2S1, 2U1, 2W1, 3A4, 4B1. Exposure to aryl hydrocarbon receptor ligands and also exposure to retinoic acid, a negative regulator of epidermal differentiation in vitro (Fisher and Voorhees 1996) led to decreases of the levels of these CYPs RNAs. On the other hand, CYP2U1 RNA level was highest in undifferentiated keratinocytes. Du et al. (2004) had reported the presence of a further CYP mRNA, CYP2R, in human keratinocytes after 6 days in culture.

Swanson (2004) observed in fetal human keratinocytes the following CYPs mRNAs: 1A1, 1A2, 1B1, 2A6, 2B6, 2C8, 3A4, 26A1. mRNA coding for CYP1A1, CYP1A2 and CYP1B1 were increased after treatment with dioxin.

*Reconstructed skin models* (overview in Table 10). mRNAs coding for CYP1A1, 1B1, 2E1, 2J2, 3A5, 4B1, low levels of RNAs coding for CYP2C18 and 3A4, but no RNA coding for CYP2B6 were recognized by Neis et al. (2010) in all of the following 3-dimensional reconstructed human skin models, the EpiDermFT^™^ full-thickness skin equivalent (MatTek, Ashland, MA, USA), the Phenion^®^ full-thickness skin model (Phenion/Henkel, Düsseldorf, Germany) and the AST-2000 (“Advanced Skin Test”) full-thickness skin model (CellSystems^®^, St. Katharinen, Germany) and in an in-house model of the authors. The CYPs mRNA levels were strongly increased by liquor carbonis detergens (except of only a weak increase of CYP1A1 mRNA in Phenion^®^).

Luu-The et al. (2009) observed in total human skin, in human epidermis, in EpiSkin^™^ and in EpiskinTMF^™^ mRNAs for the following CYPs: Low levels (below 200,000 copies/µg total RNA): CYP2B6, 2D6, 2E1, 1A1, 1B1, 2C8, 2C18, 2F1 and 3A5; very low levels (below 10,000 copies/µg total RNA): CYP2C9, 1A2 and 3A7.

Hu et al. (2010) found a high concordance (83–86%) of xenobiotic-metabolizing enzymes mRNA levels between FTHBS (full-thickness human bottom skin) and EpiDerm^™^ with the exception of mRNAs for CYP1A1, 2A6, 2E1, 4 × 1 and 8B1 which were observed in FTHBS, but not in EpiDerm^™^. mRNAs for CYP2D6, 2U1 and 2W1 were present in FTHBS, but variable between individual EpiDerm^™^ samples. A considerable number of CYPs mRNAs were found in both, FTHBS and EpiDerm^™^: CYP1A2, 1B1, 2C9, 2C18, 2J2, 2R1, 2S1, 3A5 and 4B1 and a considerable number of CYPs mRNAs were found in neither FTHBS nor EpiDerm^™^: CYP2B6, 2C8, 2C19, 2F1, 3A4 and 3A7 (for further studies on CYPs transcripts expression in reconstructed human skin models see Hayden et al. 2006; Rassmussen et al. 2011; Wiegand et al. 2008).

Very recently Bacqueville et al. (2017) reported the following on the individual expression of CYP mRNAs in their ORS-RHE model (Guiraud et al. 2014): The major hepatic CYPs, namely CYP1A2, CYP2C9/18/19 and CYP3A4 were absent or present only at low levels, notably CYP1A2, CYP2B6, CYP3A4 and the fetal form CYP3A7 were not expressed, while the following were expressed at weak or good levels: CYP1B1, CYP2C18, CYP2R1, CYP2S1, CYP3A5, CYP4B1, CYP4F2, CYP4F3, CYP4F11, CYP7B1, CYP26B1 and CYP27B1. The expression of CYP2D6 was very low or nil. CYP1A1 which was expressed at very low levels, was dramatically induced by βNF (133-fold) and 3-MC (250-fold), while CYP1B1 was more moderately induced (14-fold by β-naphthoflavone and 12-fold by 3-MC).

#### CYP protein expression

Pendlington et al. (1994) reported the presence of CYP1A1/A2 and CYP2B1/B2 proteins in human skin, localized predominantly in the epidermis and sebaceous glands. A differential localization of individual CYP proteins was observed by Katiyar et al. (2000) in that CYP1A1 protein was found predominantly in the basal cells of the (buttock) epidermis, but CYP1B1 protein in the epidermal cells other than the basal cells. Exposure to UVB led to an increase of epidermal CYP1A1 and 1B1 mRNA. In partial contrast to the above described localization study Baron et al. (2001) observed CYP1A1, 2B6, 2E1 and 3A protein (in foreskin) predominantly localized in the suprabasal layer of the epidermis.

Costa et al. (2010) showed in human whole skin increases of CYP1A1 protein after pretreatment with benzo[*a*]pyrene.

In untreated proliferating human foreskin keratinocytes in culture Baron et al. (2001) demonstrated the presence of CYP1A1, 2B6, 2E1 and 3A proteins, CYP1A1 protein at least in some individual cells. Treatment with benz[*a*]anthracene led to a large increase in the number of cells containing CYP1A1 protein and to a large increase of the level of this protein.

In an intriguing contrast to these early studies, more recent studies report that they did not find CYPs proteins including all major xenobiotica-metabolizing CYPs. The study by van Eijl et al. (2012) used proteomics and was based on custom-built PROTSIFT software monitoring in whole skin the presence of at least two tryptic peptides in at least two individual females of a mean age of 44 ± 13 years. 13 CYP proteins including all major xenobiotica-metabolizing CYPs were not detected (LOD 0.1–0.2 pmol/mg microsomal protein). CYP1A2, 2E1 and 3A4 proteins were additionally sought and not found by immunoblotting (LOD 2.5 pmol/mg microsomal protein). Likewise Hewitt et al. (2013) reported that they did not find CYP1, CYP2B6 or CYP3A proteins In the reconstructed human skin models EpiDerm, EpiSkin^™^ and SkinEthik^™^ RHE.

Future studies may resolve whether the earlier studies erroneously recognized proteins which in reality were not CYPs or whether the more recent studies failed to recognize CYP proteins in reality being present in the human skin at sufficient levels to be recognized.

#### CYP catalytic activities

See also Tables 1, 8 and 9.

**Table 8:** Representative (more examples and references in the text) Cytochrome P450 (CYP) activities [constitutive; number after slash: induced (highest reported induced activity)] in human skin and human cells in culture. Reproduced from Oesch et al. (2014)

Activity (preferential for)	Skin microsomes	Keratinocytes^a^	HaCaT	NCTC 2544	KeratinoSens^®^	LuSens	U937	THP-1
AHH (CYP1 family)	0.24–1.35^b^							
EROD (CYP1 family)	bd–35^b^	bd–10.7^b^/19^b^	bd–0.047^b^–21.9^c^/253 ± 35^c^	0.2–0.3^b^; 2 ± 0.1^d^/114 ± 77^b^	bd	bd	bd	bd
MROD (CYP1A2)	bd to +		?/35^b^	?/70^b^				
PROD (CYP2B6)	bd to bq	bd–1.43^b^/2.7^b^	bd/bd	bd–0.6 ± 0.05^d^	bd	bd	bd	bd
MFCOD (CYP1/2, especially 2C9)	bd							
BROD (CYP2B/3A)					bd	bd	bd	bd
Tolbutamide 4-hydroxylation (CYP2C9)	0.46 ± 0.05^c^	bd						
Bufuralol 1-hydroxylation (CYP2D6)	bd		bd					
Chlorzoxazone 6-hydroxylation (CYP2E1)	2.83 ± 0.34^c^		bd					
Para-nitrophenol hydroxylation (CYP2E1)	bd/+	1810^b^						
Midazolam 1-hydroxylation (CYP3A4)	2.35 ± 0.23^c^		bd					
BQOD (CYP3A)	bd–76 ± 41^b^	bd/bq	38 ± 13^b^	33 ± 5^b^				
Erythromycin *N*-demethylase (CYP3A) +	3260^b^							

For description of the cells in culture see Table 7

*AHH* aryl hydrocarbon hydroxylase, phenolic benzo[*a*]pyrene metabolites determined with 3-hydroxybenzo[*a*]pyrene as standard, *bd* below detection, *BROD* 7-benzyloxyresorufin *O*-debenzylase, *BQOD* benzyloxyquinoline *O*-dealkylase, *ECOD* 7-ethoxycoumarin-*O*-dealkylase, *EROD* 7-ethoxyresorufin-*O*-dealkylase, *MFCOD* 7-methoxy-4-trifluoromethylcoumarin *O*-dealkylase, *MROD* 7-methoxyresorufin-*O*-deethylase, *PROD* 7-pentoxyresorufin *O*-dealkylase

^a^Primary keratinocytes in culture (“NHEC”, “Normal Human Epithelial Keratinocytes”)

^b^pmol/min/mg microsomal protein

^c^pmol/h/mg microsomal protein

^d^pmol/min/mg protein determined in culture medium

**Table 9:** Representative (more examples and references in the text) Cytochrome P450 (CYP) activities [constitutive; number after slash: induced (highest reported induced activity)] in human skin and human reconstructed skin models

Activity (preferential for)	Skin	EpiDerm^™^	Episkin^™^	Episkin^™^ FTM	SkinEthik^™^ RHE	Phenion^®^ FT	StrataTest^®^	ORS-RHE
AHH (CYP1 family)	0.24–1.35^a^							
EROD (CYP1 family)	bd–35^a^ 3.0 ± 1.2^d^	bd/1.7 ± 0.8^c^	7.8 ± 0.4^d^	2.6 ± 0.3^d^	2.8 ± 0.9^d^	bd	< 1^a^	+
MROD (CYP1A2)	bd to +	bd/0.7 ± 0.3^c^						
PROD (CYP2B6)	bd to bq	bd/bd				bd	< 4^a^	
ECOD (CYP1A,1B,2B,2D6,3A4)								+
MFCOD (CYP1/2, especially 2C9)	bd ≤ ~ 0.5^d^	bd/bd	≤ ~ 0.5^d^	≤ ~ 0.5^d^	≤ ~ 0.5^d^			
BROD (CYP2B/3A)		bd				bd	< 1^a^	
Tolbutamide 4-hydroxylation (CYP2C9)	0.46 ± 0.05^b^							
Bufuralol 1-hydroxylation (CYP2D6)	bd							
Chlorzoxazone 6-hydroxylation (CYP2E1)	2.83 ± 0.34^b^							
Para-nitrophenol hydroxylation (CYP2E1)	bd/+							
Midazolam 1-hydroxylation (CYP3A4)	2.35 ± 0.23^b^							
BQOD (CYP3A)	bd–76 ± 41^a^	94 ± 13^a^/5.5 ± 0.9^c^						
Erythromycin *N*-demethylase (CYP3A)	+							
Testosterone androsterone formation (CYP2C18/19)								+
Testosterone 7α-hydroxylation (CYP2A6/2A13)								+
Testosterone 6β-hydroxylation (CYP1A1/2, CYP3A4/5/7, CYP3A43)								+
Testosterone 16α-hydroxylation								+

For description of the reconstructed human skin models see Table 10

*AHH*, aryl hydrocarbon hydroxylase, phenolic benzo[*a*]pyrene metabolites determined with 3-hydroxybenzo[*a*]pyrene as standard, *bd* below detection, *BROD* 7-benzyloxyresorufin *O*-debenzylase, *BQOD* benzyloxyquinoline *O*-dealkylase, *ECOD* 7-ethoxycoumarin-*O*-dealkylase, *EROD* 7-ethoxyresorufin-*O*-dealkylase, *MFCOD* 7-methoxy-4-trifluoromethylcoumarin *O*-dealkylase, *MROD* 7-methoxyresorufin-*O*-deethylase, *PROD* 7-pentoxyresorufin *O*-dealkylase

^a^pmol/min/mg microsomal protein

^b^pmol/h/mg microsomal protein

^c^pmol/min/mg intact model protein

^d^pmol of products/6 h/mg protein (sum of 7500 g supernatant + medium)

##### Aryl hydrocarbon hydroxylase (AHH)

The term aryl hydrocarbon hydroxylase is usually used to denote the enzymatic activity which converts benzo[*a*]pyrene to its hydroxylated metabolites. Already early on Levin et al. (1972) found in human neonatal foreskin cultured for 16 h low AHH activity (2–4 pmol hydroxylated BP/mg protein in 30 min) which was increased by treatment with benz[*a*]anthracene. Alvares et al. (1973a, b) presented the following indications that this activity was produced by CYP: requirement for molecular oxygen and for NADPH, complete inhibition by carbon monoxide and a pH optimum of 7.4. Merk et al. (1987a) confirmed that this AHH activity (of human hair follicles) required NADPH and was inhibited by carbon monoxide and also by the CYP inhibitors ketoconazole and alpha-naphthoflavone.

Remarkably, hair follicles had higher AHH activities compared with total skin (Merk et al. 1987a) and AHH activity was reduced in psoriatic skin lesions, but not in the surrounding skin (Shuster et al. 1980; Bickers et al. 1984).

In skin tape strip samples from naphthalene-exposed workers naphthyl-keratin adducts were seen Kang-Sickel et al. (2010), in all likelihood resulting from AHH-mediated activation of naphthalene to protein-reactive metabolite(s).

The prototypic substrate of AHH, BP, is converted in primary human keratinocyte cultures (“normal human epithelial keratinocytes”; NHEK) to the immediate AHH metabolites, hydroxy-BPs, as well as multistep generated metabolites including diols and BP-7,8,9,10-tetraol, the latter indicating that the ultimate carcinogen (+)-anti-BP-7,8-diol-9,10-epoxide (BPDE) had been transiently formed. In primary human skin-derived fibroblast cultures the total amounts of BP-derived metabolites were similar as in the NHEK, but the amounts of BP-7,8,9,10-tetraol was lower and that of the BP-7,8-diol was higher compared with the NHEK, indicating that fibroblasts generate lower amounts of the ultimate carcinogen BPDE. The reconstructed full-thickness human skin model MatTek EpiDermFT and the human epidermal skin model MatTek EpiDerm provided a good accordance in BP metabolism compared with human skin, whereas confluent NHEK yielded 32.5-fold less BP metabolites than did human skin (Brinkmann et al. 2013).

##### Prototypical CYP catalytic activities other than AHH

*Human skin* In contrast to rat and mouse skin homogenates (investigated in the same study) no 7-ethoxycoumarin-*O*-dealkylase (ECOD) activity (CYP1A and 2B-selective) was detected in human skin homogenate (Damen and Mier 1982). On the other hand, Merk et al. (1987b) observed in human hair follicles ECOD activity, which was increased by topical application of liquor carbonis detergens containing CYP-inducing PAHs and was inhibited by the CYP inhibitor ketoconazole. The finding of ECOD activity in the human skin was confirmed by Ademola et al. (1993).

The following prototypic CYP activities were found (or not found, respectively) in human skin microsomes by Rolsted et al. (2008): EROD (CYP1-selective) not detected (LOQ 1.87 pmol), PROD (CYP2B-selective) not detected (LOQ 1.87 pmol), tolbutamide 4-hydroxylation (CYP2C9-selective) 0.46 ± 0.05 pmol/h/mg protein, bufuralol 1-hydroxylation (CYP 2D6-selective) not detected (LOQ 1.08 pmol), chlorzoxazone 6-hydroxylation (CYP2E1-selective) 2.83 ± 0.34 pmol/h/mg protein, midazolam 1-hydroxylation (CYP3A-selective) 2.35 ± 0.23 pmol/h/mg protein.

Götz et al. (2012a) noticed in human skin microsomes very low CYP3A activities (just exceeding the LOD) using 7-benzyloxyquinoline (BQ) as selective CYP3A substrate and using the luminescent Luc-BE CYP3A-assay. EROD, 7-methoxyresorufin-*O*-demethylase (MROD), PROD or *O*-dealkylase activities for 7-methoxy-4-trifluoromethylcoumarin (broad-spectrum CYP1⁄2) substrate were not detected.

Similar to this two other studies (Jäckh et al. 2011; Rolsted et al. 2008) report the failure of EROD detection above the LOQ (2 pmol/min/mg protein and 1.87 pmol, respectively) in human skin microsomes.

Du et al. (2009) reported the observation of microsomal leukotriene B4 hydroxylation activity in human full-thickness skin biopsies and its increase by treatment with retinoic acid.

*Cell and organ culture* (for cell lines see overview in Table 7). In human keratinocytes in culture Raffali et al. (1994) observed ECOD activity. The CYP inducers and inhibitors miconazole and econazole at low concentrations led to an increase and at high concentrations to an inhibition of the ECOD activity. Clotrimazole and sulconazole led only to an increase of ECOD activity, imidazole did not lead to a change of ECOD activity.

In human keratinocytes submerged cultures EROD activities were present, but decreased by 65% after confluency, while phenacetin deethylase activities were only slightly decreased (Hirel et al. 1995). No differences were noted between keratinocytes in primary culture versus secondary subcultures. EROD activity was increased in presence of 3-MC and in presence of benz[*a*]anthracene. Both activities (EROD and phenacetin deethylase) were not markedly changed after a freezing/thawing cycle, whereas Kao et al. (1985) reported a drastic decrease of benzo[*a*]pyrene and testosterone metabolism after a freezing/thawing cycle of whole skin organ cultures. EROD and phenacetin deethylase activities were not markedly different in fresh versus viable human keratinocytes obtained 24–50 h after death (Hirel et al. 1996).

Baron et al. (2001) reported the following activities determined in microsomes obtained from cultured human keratinocytes: EROD (CYP 1-selective): 10.7 pmol/min/mg protein, PROD (CYP2B-selective): 1.43 pmol/min/mg protein, para-nitrophenol hydroxylase (CYP2E1-selective): 1.81 nmol/min/mg protein, erythromycin *N*-demethylase (CYP 3A-selective): 3.26 nmol/min/mg protein.

In the human keratinocyte-derived cell line HaCaT (Boukamp et al. 1988) EROD activity was present and increased by the CYP1 inducer 3-MC (Delescluse et al. 1997). The following activities and lack of detectable activities, respectively, were observed by Rolsted et al. (2008) in HaCat cells: EROD activity: 21.9 ± 1.9 pmol/h/mg protein, after treatment with with beta-naphthoflavone 253 ± 35 pmol/h/mg protein. A comparison of HaCaT and NHEC showed in HaCaT EROD activities near the LOD (0.047 “pmol/mg/min”), in NHEK between 0 and 0.76 pmol/min/mg protein, in both of these cell types highly increased by treatment with the CYP1 inducer BP (Bonifas et al. 2010a, b).

In the human skin keratinocyte-derived cell line NCTC 2544 (Bakken et al. 1961) ECOD, EROD and PROD activities were present and were increased by treatment with the CYP inducers 3-methylcholanthrene, β-naphthoflavone and phenobarbital and decreased by the presence of the CYP inhibitors α-naphthoflavone and metyrapone (Gelardi et al. 2001).

A comparative study by Götz et al. (2012a) showed EROD activity for NHEK and NCTC 2544 just above the LOD at 0.2–0.3 pmol/min/mg protein, while in HaCaT, no activity was detected. Treatment with 3-methylcholanthrene led to an increase of EROD (CYP1-selective) and MROD (CYP1A2-selective) which was higher in the immortalized cell lines HaCaT and NCTC 2544 than in the primary keratinocytes NHEK. PROD activity (CYP2B-selective) was not increased by rifampicin, CITCO (6-(4-chlorophenyl)imidazo-[2,1-*b*][1,3]thiazole-5-carbaldehyde-*O*-(3,4-dichlorobenzyl)oxime) or cyclophosphamide.

Fabian et al. (2013) reported that in U937 und THP-1 (dendritic cell lines from the German Collection of Microorganisms and Cell Cultures) and in the keratinocytic cell lines LuSens (developed by BASF SE) and KeratinoSens^®^ (developed by Givaudan) EROD, PROD and BROD activities were below the LOD.

*Reconstructed skin models* (overview in Table 10). EROD, PROD and 7-benzoxyresorufin *O*-debenzylase activities were observed already early on in a reconstructed epidermis model generated from human hair follicles of the outer root sheath (Pham et al. 1990). ECOD activity was seen in EpiSkin^™^ and was increased at low concentrations of the CYP inducers and inhibitors econazole and clotrimazole, and was inhibited in presence of their high concentrations (Cotovio et al. 1996). Harris et al. (2002a) observed that EROD was induced by 3-methylcholanthrene in 5 of 6 batches of EpiDerm^™^, in 2 of 5 batches EpiSkin^™^ and in 2 of 6 batches SkinEthic^™^, but was not detected constitutively. ECOD activity was constitutively present and increased upon treatment with 3-methylcholanthrene in the same batches in which EROD was increased. Hayden et al. (2006) and Hu et al. (2010) reported a pronounced increase in EROD activity in EpiDerm^™^ by treatment with 3-methylcholanthrene. In EpiDerm^™^ and Phenion^®^FT (PFT) the investigated CYP activities were not detected, whereby the LODs were, respectively, for EROD 0.001 nmol/min/mg protein, for PROD 0.0025 nmol/min/mg protein and for BROD 0.002 nmol/min/mg protein (Jäckh et al. 2011). In EpiDerm^™^ microsomes no EROD, MROD, PROD or 7-methoxy-4-trifluoromethylcoumarin *O*-dealkylase activities were detected (LOD for EROD, MROD and PROD < 0.2 pmol⁄min⁄mg protein), while very low CYP3A activities were detected (Götz et al. 2012a). These activities and lack of observed activities, respectively, were similar as the results obtained by the same authors in human skin (presented above under “Human skin”). In intact EpiDerm^™^ EROD and PROD activities were increased by treatment with 3-methylcholanthrene, while PROD and 7-methoxy-4-trifluoromethylcoumarin *O*-dealkylase activities remained undetected even after treatment with CITCO (6-(4-chlorophenyl)imidazo[2,1-*b*][1,3]thiazole-5-carbaldehyde-*O*-(3,4-dichlorobenzyl)oxime) or rifampicin. Guth (2013) reported that in the human epidermal skin model StrataTest^®^ she did not detect EROD, PROD or BROD activities (LOD 1, 4 and 1 pmol/min/mg protein, respectively). On the other hand, measurable dealkylase activities were recently reported as pmol/mg protein/6 h; sum of S9 + medium:

- EROD in SkinEthik^™^ RHE: 2.8, in Episkin^™^: 9.1, in Episkin^™^ FTM: 2.6, compared with human skin: 3.0;
- 7-benzoxy 4-trifluoromethylcoumarin *O*-deethylase activities (CYP3A5/3A7-selective) in SkinEthik^™^ RHE: 13.3, in Episkin^™^: 3.6, in Episkin^™^ FTM: 3.3, compared with human skin: 3.8;
- 7-methoxy-4-trifluoromethylcoumarin *O*-dealkylase activity was close to the LOQ of 0.5 in all three skin models and in human skin.

**Table 10:** Overview of commercially available reconstructed human skin models. Adapted from: Jäckh et al. (2012)

Skin model	Type	Company	Cell origin	References
EpiDerm^™^ (EPI-200)	Epidermal	MatTek, MA, USA	Male foreskin	Hayden et al. (2006)
EpiDermFT^™^ (EFT)	Full-thickness (collagen matrix)	MatTek, MA, USA	Male foreskin	Hayden et al. (2003)
EST-1000 (EST)	Epidermal	Cell Systems, Troisdorf, Germany	Male foreskin	Hoffmann et al. (2005)
AST-2000 (AST)	Full-thickness (collagen matrix)	Cell Systems, Troisdorf, Germany	Male foreskin	Hoffmann et al. (2003)
Episkin^™^	epidermal	SkinEthic^™^ laboratories, Nice, France	Adult breast skin	Tinois et al. (1991) Netzlaff et al. (2005)
Episkin^™^ FTM	Full-thickness (polycarbonate and collagen matrix)	SkinEthic^™^ laboratories	Adult breast skin	Eilstein et al. (2010)
SkinEthik^™^ RHE	Epidermal	SkinEthic^™^ laboratories, Nice, France	Adult abdomen Male foreskin	Netzlaff et al. (2005) Eilstein et al. (2014)
Phenion^®^ FT (PFT)	Full-thickness (collagen matrix)	Henkel, Düsseldorf, Germany	Male foreskin	Mewes et al. (2007)
StrataTest^®^	Epidermal	StrataTech, MA, USA	NIKS^®^ human keratinocyte cell line	Slavik et al. (2007)
EuroSkin^®^	Epidermal	EuroDerm, Leipzig, Germany	Hair follicles	http://www.eurodermbiotech.de (2011)
EPI-MODEL	Epidermal	LabCyte, Aichi, Japan	?	Katoh et al. (2009)
OS-REp	Epidermal	Henkel, Düsseldorf, Germany	Cultured human keratinocytes	Poumay et al. (2004)
ORS-RHE	Epidermal	PierreFabreDermo-cosmétique	Outer root sheath	Guiraud et al. (2014)

*NIKS®* Spontaneously Immortalized Near-Diploid Human Keratinocyte Cell Line

Very recently Bacqueville et al. (2017) reported EROD activity and its induction by 3MC and by beta-naphthoflavone in their ORS-RHE model (Guiraud et al. 2014). They observed the following metabolites produced from testosterone by their ORS-RHE model: androsterone, one of the major metabolites [the production of which is catalyzed by CYP2C18/19 (Yengi et al. 2003)], 7α-hydroxytestosterone [produced by CYP2A6 and 2A13 (Bogaards et al. 2000; Desille et al. 1999)], 6β-hydroxytestosterone [produced by CYP1A1/2, CYP3A4/5/7 and CYP3A43 (Soucek et al. 2001; Waxman et al. 1991)], a reduced metabolite, 5α-androstan-3,17-dione [catalyzed by 5α-reductase (Liu and Yamauchi 2008)] as well as epiandrosterone, androstenedione, Δ6-testosterone, hydroxy-Δ4-dione, 4-androsten-16α-ol-3,17-dione and 16α-hydroxytestosterone. BP was metabolized by ORS-RHE to hydroxy-, diol, catechol and dione metabolites (known to be mainly produced by CYP1B1, CYP1A1 and microsomal epoxide hydrolases (Hvastkovs et al. 2007; Oesch and Arand 1999b] and in part by CYP2C9, and CYP3A48; (Gautier et al. 1996)].

#### Individual CYPs

##### CYP1 family

Family 1 CYP transcripts, proteins and enzymatic activities in the human (and rodent) skin have been shown to be drastically increased by treatment with many planar polyhalogenated dibenzo-para-dioxins, polyhalogenated dibenzofurans, polycyclic aromatic hydrocarbons as well as co-planar polyhalogenated byphenyls (Levin et al. 1972; Alvares et al. 1973a, b; Mukhtar and Bickers 1983; Finnen et al. 1984; Whitlock 1987; Merk et al. 1987a; Vecchini et al. 1995). These drastic increase are mediated (at least in great part) by the *Ah receptor* (AhR), which, especially in immune cells of the skin (and of the intestine), is counterregulated by the AhR repressor (AhRR) (Brandstätter et al. 2016). The concentration of AhR is highest in the spinous and in the granular layers and lowest in the basal layer of the human skin epidermis (Swanson 2004). However, almost all cell types present in the skin including many immunologically important cell types express the AhR. In the epidermis keratinocytes, melanocytes, and Langerhans cells express functional AhR. In the dermis fibroblasts, dendritic cells, Th17 cells, and γ,δ-T cells of the IL-17-producing subsetVγ2 express AhR (Stockinger et al. 2014 and references therein). Fritsche et al. (2007) observed that extremely low concentrations (low picomolar) of the AhR ligand 6-formylindolo[3,2-*b*]carbazole (FICZ) are sufficient to cause significant increases of the CYP1A1 transcript. This potent AhR activator FICZ and several other indole-related AhR activators are derived from the essential amino acid tryptophan via the enzymes indolamine 2,3-dioxygenase (IDO) and tryptophan 2,3-dioxygenase (TDO). UV light converts tryptophan to derivatives with very high affinity for the AhR (Rannug et al. 1987). In addition, indole-related AhR activators can stem from dietary intake including cruciferous vegetables, such as Brassica, which are rich in the glucosinolate glucobrassicin that is enzymatically degraded to indole-3-carbinol (I3C), and then further converted to the high-affinity AhR ligands indolo-(3,2-*b*]-carbazole (ICZ) and 3,3′-diindolylmethane in the presence of ascorbic acid under acidic conditions in the stomach (Stockinger et al. 2014 and references therein). Very recently Smirnova et al. (2016) reported that the yeast Malassezia which constitutes a part of the normal skin microbiota, produces this potent AhR activator FICZ as well as several further AhR activators including indolo[3,2-*b*]carbazole-6-carboxylic acid. In addition to this they produced strong suggestive evidence that FICZ is likely to be formed systemically (in humans and rodents). Berghard et al. (1990) reported that induction of CYP1A1 transcript by 2,3,7,8-tetrachlorodibenzofuran (TCDF) occurred in human keratinocytes in culture at an EC50 of 2 nM, but only in presence of either serum or high calcium (2 mM) concentration. Since these conditions were similar to those required for induction of the transcript coding for the differentiation-specific epidermal transglutaminase it appears that terminal differentiation is a prerequisite for CYP1A1 induction by AhR ligands to happen in human skin. Interestingly, recent studies by Borland et al. (2014) showed that the AhR-dependent induction of the expression of several CYPs (as well as several phase II xenobiotic-metabolizing enzymes) in human keratinocytes required the presence of peroxisome proliferator-activated receptor β/δ (PPARβ/δ). They compared Pparβ/δ-knock down with wild-type human HaCat keratinocytes (and also Pparβ/δ null mice primary keratinocytes as well as whole animals versus their wild types) which showed an intriguing cross talk between PPARβ/δ and AhR signaling and consequent inductions of CYP mRNA expression (and expression of phase II xenobiotic-metabolizing enzymes). Vorrink et al. (2014) reported that the occurrence of the CYP1 inducer PCB 126 (3,3′,4,4′,5-pentachlorobiphenyl)-evoqued increase in CYP1A1 mRNA and protein expression was inhibited by hypoxia. Ledirac et al. (1997) reported that mechanisms other than ligand-binding to AhR-dependent events must exist which also lead to CYP1 induction, since carbaryl induced CYP1A1 expression in HaCaT without being an AhR ligand. Physiologically CYPs of the CYP1 family have been found (in the HaCaT keratinocyte cell line) to be required for the normal cell cycle (Gundert-Remy et al. 2014 and references therein) and presence versus absence of AhR plays a complex cell proliferation inducing and inhibiting role, in part dependent on the cell types in question (Weiss et al. 2008; Stockinger et al. 2014 and references therein).

*Cyp1A1 mRNA* was variably expressed in native human skin, both in the epidermis (moderate expression in the epidermis of two out of three donors) and in the dermis (weak but significant expression in the dermis from two out of three donors). In monolayer epidermal cells (NHEK) and in monolayer fibroblasts CYP1A1 mRNA was weakly, but significantly expressed in cultures derived from all three above-mentioned donors. CYP1A1 mRNA was moderately expressed in the three-dimensional epidermal reconstructed model OS-REp from all three above-mentioned donors, but in none of the three-dimensional full skin model Phenion FT Skin (Wiegand et al. 2014). The latter discrepancy between mRNA expression in Phenion FT Skin versus native human skin is in contrast to the expression of mRNAs coding for most other xenobiotica-metabolizing enzymes which are quite well-mirrored in this three-dimensional full skin model, while the mRNA expression in the epidermal three-dimensional model OS-REp also compared quite well with that of the epidermal compartment of the native skin (see in the following chapters).

*CYP1A1 protein* Polycyclic aromatic hydrocarbons, such as BP, dibenzo[*a,l*]pyrene, 7,12-dimethylbenz[*a*]anthracene, and other PAH are substrates of CYP1A1 generating electrophilically reactive genotoxins including epoxides, but also phenols which are then conjugated yielding glucuronides and sulfates allowing for facilitated excretion. As a consequence of this dual role modulations of CYP1A1 activities, e.g., by induction may lead to opposite effects after different routes of administration depending whether these lead first-pass effects or to direct exposure of target tissues (Nebert et al. 2004). Schober et al. (2006) reported that metabolic activation of dibenzo[*a,l*]pyrene (as far as hitherto known the most carcinogenic PAH) by heterologously expressed CYPs is most efficient with human CYP1A1 as compared to other human CYPs and as compared to any rat CYP.

*CYP1A2* The following arguments indicate that CYP1A2 is present in the human skin: several enzymatic activities which are typical for CYP1A2 have been observed in human skin, including MROD as well as theophylline demethylation (Ademola et al. 1993). Schober et al. (2006) reported that metabolic activation of dibenzo[*a,l*]pyrene is catalyzed by heterologously expressed CYP1A2, albeit not as well as by human CYP1A1 or by CYP1B1, but better than by other human CYPs.

Other CYP1A2 substrates (as observed in skin or in other systems) include aflatoxin B1, aromatic amines and aromatic amides, caffeine, acetaminophen, phenacetin, imipramine, warfarin (Du et al. 2004; Oesch-Bartlomowicz and Oesch 2007).

*CYP1B1* PAH toxication to ultimate carcinogens is efficiently mediated also by CYP1B1. PAH as well as their dihydrodiols are important substrates of CYP1B1 (Oesch-Bartlomowicz and Oesch 2007). Schober et al. (2006) showed that metabolic activation of the most carcinogenic member of the PAH, dibenzo[*a,l*]pyrene, is efficiently catalyzed by heterologously expressed human CYP1B1, much better than by other human CYPs and by rat CYPs, but less efficiently than by human CYP1A1. Sutter et al. (1994) showed that CYP1B1 was constitutively expressed and highly inducible in primary human keratinocytes, which by Sutter et al. (1994) and Akintobi et al. (2007) was also observed in human fibroblasts. Villard et al. (2002) reported that in primary human keratinocytes as well as in HaCaT cell cultures the expression of CYP1B1 on the mRNA level was increased by UVB irradiation to interindividually largely varying degrees (1.1- to 4.5-fold). The increase was AhR-dependent and was inhibited by the antioxidant *N*-acetylcysteine. Based on these observations the authors suggested that the formation of UVB photoproducts was involved in the induction.

Several reports showed cell-type selectivity of CYP1B1 presence and induction. Merk (2009) reported that carvoxime induced CYP 1B1 in dendritic cells, but not in keratinocytes. Murray et al. (1997) showed a strong immunoreactivity to highly specific anti CYP1B1 antibodies (no cross reactivity with CYP1A) in human skin cancer, but not in normal human skin. Katiyar et al. (2000) observed CYP1B1 protein in healthy human skin, but localized exclusively in epidermal cells other than those in the basal layer. In a recent systematically comparative study of Wiegand et al. (2014) Cyp1B1 mRNA was consistently and moderately to highly expressed in native human skin, both in the epidermis and in the dermis, in monolayer epidermal cells (NHEK) and fibroblasts and in the three-dimensional full skin model Phenion FT Skin and in the three-dimensional epidermal reconstructed model OS-REp (Wiegand et al. 2014). Most recently Bacqueville et al. (2017) reported that in their three-dimensional ORS-RHE model 7-ethoxycoumarin was efficiently deethylated. Since basal CYP2E1 expression was absent in these ORS-RHE models, they concluded that the CYP responsible for the biotransformation of 7-ethoxycoumarin to 7-hydroxycoumarin was likely to be CYP1B1, which they had found to be expressed at good levels.

##### CYP2 family

mRNAs coding for the following CYPs were detected in the human skin by Du et al. (2004): CYP2A6, 2A7, 2B6, 2C9, 2C18, 2C19, 2D6, 2E1, 2J2, 2R1, 2S1, 2U1, 2W1.

*CYP2A subfamily* Janmohamed et al. (2001) reported the finding of mRNA coding for *CYP2A6* in human skin, but neither in proliferating keratinocytes in culture nor in HaCaT. They concluded that mRNA coding for CYP2A6 is produced either in terminally differentiated, not proliferating keratinocytes or in the dermis. Ding et al. (1995) reported the finding of mRNA coding for *CYP2A7* in human skin fibroblasts, but upon heterologous expression of CYP2A7 they did not observe enzymatic activity. No expression of *CYP2A13* was seen in the human skin. In a recent systematically comparative study of Wiegand et al. (2014) Cyp2A6 mRNA was very variable. It was variably expressed in native human skin (moderate expression in the epidermis of two out of three donors and weak expression in the dermis of one out of three donors). In monolayer epidermal cells (NHEK) CYP2A6 mRNA was not expressed and in monolayer fibroblasts CYP2A6 mRNA was weakly expressed in cultures derived from one of the three above-mentioned donors. CYP2A6 mRNA was weakly expressed in the three-dimensional epidermal reconstructed model OS-Rep derived from all three above-mentioned donors and in the three-dimensional full skin model Phenion FT Skin CYP2A6 mRNA was weakly expressed in the epidermis of one of the three donors and in the dermis of two of the three donors (Wiegand et al. 2014).

The substrate specificity of CYP2A6 protein (investigated in organs other than the skin) includes:

- physiological compounds (e.g., progesterone, retinoic acid, arachidonic acid) and
- xenobiotics (e.g., 6-aminochrysene, several nitrosamines, aflatoxin B_1_, coumarin, nicotine) (Oesch-Bartlomowicz and Oesch 2007).

*CYP2B subfamily* Janmohamed et al. (2001) and Hu et al. (2010) reported the finding of *CYP2B6 mRNA* in human skin, Baron et al. (2001) in proliferating human keratinocytes in culture and in HaCaT cells. In contrast to these positive findings Saeki et al. (2002) did neither detect CYP2B6 mRNA in human skin nor in primary human keratinocytes nor in fibroblasts nor in Langerhans cells. Likewise Rolsted et al. (2008) and Rassmussen et al. (2011) did not detect CYP2B6 mRNA in the three-dimensional reconstructed human skin models EpiDerm^™^ and StrataTest^®^. In a recent systematically comparative study of Wiegand et al. (2014) Cyp2B6 mRNA was not detected in native human skin, neither in the epidermis nor in the dermis. It was neither detected in monolayer epidermal cells (NHEK) nor in fibroblasts nor in the three-dimensional full skin model Phenion FT Skin nor in the three-dimensional epidermal reconstructed model OS-REp (Wiegand et al. 2014). These apparently contradictory results may be resolved by the observation of Yengi et al. (2003) that CYP2B6 mRNA levels ranged in skin biopsies from 27 individuals from the highest values measured for 10 CYPs down to undetected.

Baron et al. (1983) reported that in the human epidermis CYP2B6 protein was detected and was localized in the suprabasal cell layers.

Most but not all studies report failure of finding CYP2B6-dependent enzymatic activities in human skin or human skin-derived preparations. Götz et al. (2012a) report that in human skin and in the reconstructed human skin model EpiDerm^™^ they did not detect PROD activity (LOD < 0.2 pmol/min/mg protein). Likewise Guth (2013) did not detect PROD activity in StrataTest^®^ (LOD 4 pmol/min/mg protein). Attempts to induce PROD activities in the human reconstructed skin model EpiDerm^™^ using quite a number of CYP2B6 inducers (phenobarbital, rifampicin, 6-(4-chlorophenyl)imidazo[2,1-*b*][1,3]thiazole-5-carbaldehyde-*O*-(3,4-dichlorobenzyl)oxime (CITCO) as well as cyclophosphamide) also failed (Götz et al. 2012a). In contrast to these negative findings Baron et al. (2001) report detection of PROD activity in proliferating human keratinocytes in culture and its induction by phenobarbital.

As found in skin-derived preparations or in other systems CYP2B6 is active toward:

- several physiological substrates (including 17β-estradiol, estrone, testosterone, retinoic acid),
- xenobiotic compounds (including coumarin, nicotine, cyclophosphamide, ochratoxin A) (Ekins and Wrighton 1999; Oesch-Bartlomowicz and Oesch 2007).

*CYP2C subfamily* In contrast to other CYPs, as pointed out above, the mRNAs coding for the major human CYP2C proteins, CYP2C9, CYP2C18 and CYP2C19 were found in the skin of all individuals investigated by Yengi et al. (2003). On the other hand, in proliferating human keratinocytes in culture CYP2C19 mRNA was found only after induction by clofibrate Gonzalez et al. (2001). In line with the failure of finding constitutive expression in proliferating keratinocytes, the localization of CYP2C mRNAs as well as CYP2C proteins is in most cases in the outer suprabasal cell layers of the epidermis, where differentiated keratinocytes are located (Du et al. 2004).

As found in skin or other systems CYP2Cs metabolize:

- many physiological compounds (CYP2C9: retinoic, arachidonic and linoleic acids, 5-alpha-androstane-3-alpha,17-beta-diol; 2C18: retinoic acid, progesterone; 2C19: progesterone, testosterone, retinal, arachidonic and linoleic acids);
- natural compounds (2C9: limonene, capsaicin, nicotine, galangin, genistein 4′-methyl ether, ochrotoxine A; 2C18: limonene; 2C19: limonene, capsaicin, nicotine, genistein 4′-methyl ether, ochratoxin A, tetrahydrocannabinol);
- pharmaceutical drugs [2C9: (S)-warfarin, tolbutamide, diclofenac, ibuprofen, indomethacin, phenytoin; 2C19: (R)-warfarin, omeprazole, pantoprazole, lansoprazole, (S)-mephenytoin, diazepam, amitryptiline] (Du et al. 2004; Oesch-Bartlomowicz and Oesch 2007).

*CYP2D subfamily* Yengi et al. (2003) reported that they found CYP2D6 mRNA in the skin biopsies of all 27 human individuals investigated, the levels of the CYP2D6 mRNA being among the highest of all 10 CYPs studied. On the other hand, Rolsted et al. (2008) reported that the CYP2D6-catalyzed metabolism of bufuralol did not reach the LOQ of 1.08 pmol. Saeki et al. (2002) reported that they observed CYP2D6 mRNA in human dermal fibroblasts, but not in proliferating human keratinocytes in culture. However, in a recent systematically comparative study of Wiegand et al. (2014) CYP2D6 mRNA was neither detected in the dermis of native human skin nor in monolayer fibroblasts derived from the same donors nor in the dermis of the three-dimensional full skin model Phenion FT Skin, but was consistently expressed in the three-dimensional epidermal reconstructed model OS-REp derived from any of the three donors. It was expressed in the monolayer epithelial cells NHEK derived from two of the three donors. It was not expressed in native human skin, neither in the epidermis nor in the dermis (Wiegand et al. 2014).

The substrate specificity of CYP2D6 protein (investigated in organs other than the skin) includes:

- physiological substrates (e.g., progesterone, testosterone, retinal, tryptoptamine),
- natural compounds (e.g., aflatoxin B_1_, nicotine, ochratoxin A, capsaicin, curcumin, emetine, genistein 4′-methyl ether, sparteine),
- pharmaceutical drugs (e.g., debrisoquine, bufuralol, propranolol, dextromethorphan, fluoxetine) (Du et al. 2004; Oesch-Bartlomowicz and Oesch 2007).

*CYP2E subfamily* CYP2E1 transcripts were noted in human keratinocytes, melanocytes, Langerhans cells and fibroblasts (Baron et al. 2001; Gonzalez et al. 2001; Saeki et al. 2002). Consistent with this, in a recent systematically comparative study of Wiegand et al. (2014) CYP2E1 mRNA was expressed in native human skin, both, in the epidermis and in the dermis. It was expressed in monolayer epidermal cells (NHEK) and in fibroblasts as well as in the three-dimensional full skin model Phenion FT Skin and in the three-dimensional epidermal reconstructed model OS-REp (Wiegand et al. 2014).

CYP2E protein was observed by Baron et al. (2001) in human skin, most of all in the suprabasal epidermis.

CYP2E1-typical enzymatic activity in the human skin was observed toward the substrates chlorzoxazone and toward para-nitrophenol in microsomes derived from the total human skin by Rolsted et al. (2008) and in proliferating human keratinocytes microsomes in culture by Baron et al. (2001), respectively.

Some examples of CYP2E1 substrates: ethanol, chlorzoxazone, benzene, 17β-estradiol, estrone, retinoic acid, fatty acids, aflatoxin B_1_, nicotine, capsaicin, curcumin, short-chain *N*-nitrosamines (Du et al. 2004; Oesch-Bartlomowicz and Oesch 2007).

*CYP2J subfamily* CYP2J2 mRNA and CYP2J2 protein was found in cultured human keratinocytes (Du et al. 2004).

Some examples of CYP2J2 substrates: arachidonic and linoleic acids, testosterone (Du et al. 2004).

*CYP2R subfamily* CYP2R transcripts were demonstrated in cultured human keratinocytes (Du et al. 2004).

Heterologously expressed human CYP2R1 displayed vitamin D 25-hydroxylase activity (Cheng et al. 2003). As Bikle and Pillai (1993) showed 1,25-dihydroxy-vitamin D induces differentiation in the epidermis. CYP2R1 may, therefore, be important for epithelial differentiation.

*CYP2U1 subfamily* CYP2U1 transcripts were demonstrated in cultured human keratinocytes (Du et al. 2004).

Heterologously expressed human CYP2U1 generated from arachidonic acid 19- and 20-hydroxyeicosatetraenoic acids. It also metabolized other medium and long chain fatty acids.

*CYP2W subfamily* CYP2W transcripts were demonstrated in cultured human keratinocytes (Du et al. 2004).

CYP2W1 substrates: benzphetamine and indole (Li et al. 2009).

*CYP2S subfamily* Smith et al. (2003b) reported that they found in the human skin *CYP2S1* gene promoter region two xenobiotic response elements, which were identical to those found in CYP1 genes, and, in addition, several retinoic acid receptor consensus half-sites.

Furthermore, Smith et al. (2003b) reported finding CYP2S1 mRNA in human skin. Saarikoski et al. (2005a) observed CYP2S1 mRNA in the epithelia of the respiratory and digestive tracts and in the skin. CYP2S1 mRNA was increased in the human skin by topical exposure to coal tar, retinoic acid or UV light. In a recent systematically comparative study of Wiegand et al. (2014) Cyp2S1 mRNA was ubiquitously expressed in all compared skin systems. It was expressed in native human skin, both, in the epidermis and in the dermis. It was expressed in monolayer epidermal cells (NHEK) and in fibroblasts as well as in the three-dimensional full skin model Phenion FT Skin and in the three-dimensional epidermal reconstructed model OS-REp (Wiegand et al. 2014).

In addition to the findings by Smith et al. (2003b) reported above, they observed CYP2S1 protein in the human skin and found it to be localized to the outermost, differentiated layers of the epidermis. The levels of CYP2S1 protein were increased by topical exposure to coal tar, retinoic acid or UV light.

Saarikoski et al. (2005b) found that CYP2S1 accepted naphthalene as substrate and Smith et al. (2003b) reported that CYP2S1 catalyzes the transformation of all-*trans*-retinoic acid to 4-hydroxy and 5,6-epoxy retinoic acid. However, Wu et al. (2006) did not detect these reactions by recombinant CYP2S1 at a LOD at < 0.05 and < 0.003 nmol product/min/nmol P450, respectively.

Fatty acid hydroperoxides generated in the skin by extensive UV irradiation can be used by cutaneous CYP2S1 as co-substrates in the oxidation of polycyclic aromatic hydrocarbons including the oxidation of BP-*trans*-7,8-dihydrodiol to the highly mutagenic and carcinogenic BP-r-7,t-8-dihydrodiol-t-9,10-epoxide (Bui et al. 2009), the reaction probably proceeding by a free radical mechanism via peroxidative and peroxygenative reactions (Bui and Hankinson 2009). CYP2S1 can oxidize several substrates using cumene hydroperoxide and hydrogen peroxide (Bui et al. 2009). Thus, CYP2S1 can contribute to the metabolism of carcinogens that come in contact with the skin by CYP-dependent, but NADPH-independent biotransformation (Slominski et al. 2014).

Wang and Guengerich (2013) reported that CYP2S1 is involved in the reductive metabolism of carcinogenic aromatic amines and heterocyclic aromatic amines. CYP2S1-mediated reduction of the hydroxylamines of 4-aminobiphenyl, 2-naphthylamine, and 2-aminofluorene took place even under aerobic conditions. CYP2S1 also reduced several nitroso and nitro derivatives of these arylamines. Thus, CYP2S1 quite likely is involved in the reductive detoxication of many activated metabolites of aromatic amines and heterocyclic aromatic amines.

##### CYP3 family

*CYP3A4 and CYP3A5* Baron et al. (2001) showed that CYP3A5, but not CYP3A4 mRNA was constitutively present in proliferating human skin keratinocytes in culture, while the expression of CYP3A4 mRNA was induced by treatment with dexamethasone. In a recent study by Wiegand et al. (2014) Cyp3A4 mRNA was very variably expressed in various skin systems, whereby it was not clear whether CYP3A4 was distinguished from CYP3A5. The authors report that CYP3A4 was expressed in native human skin, both in the epidermis (moderate expression in the epidermis of all three donors investigated) and in the dermis (weak expression in the dermis from one out of the three donors). In monolayer epidermal cells (NHEK) and in monolayer fibroblasts CYP1A1 mRNA was not expressed in cultures derived from any of the three above-mentioned donors. CYP1A1 mRNA was weakly to moderately expressed in the three-dimensional epidermal reconstructed model OS-REp derived from any of the three donors, but in the three-dimensional full skin model Phenion FT Skin only weakly and only in one of the three donors (Wiegand et al. 2014).

Baron et al. (2001) showed that CYP3A protein was present in human skin.

Gibbs et al. (2007) observed that in human skin testosterone was transformed to 6-β-hydroxytestosterone, a CYP3A-selective activity.

Recently Wiegand et al. (2014) reported that native human skin had a low level of CYP3A4 protein, moderately induced after 24 h by 2 mM phenobarbital, which, however, was not accompanied by an increase in CYP3A4-dependent BROD activity by phenobarbital (0.5–2 mM for 24, 48 and 72 h) nor by rifampicin (50 and 100 µM for 24 h) nor by dexamethasone (10 and 50 µM for 24, 48 and 72 h). Baron et al. (2001) observed in proliferating human skin keratinocytes in culture constitutive demethylation of the CYP3A-selective substrate erythromycin which was increased by treatment with dexamethasone. Vickers et al. (1995) reported that the hydroxylation of the CYP3A-selective substrate cyclosporine A was predominantly occurring in human dermis-derived cultures.

Some examples of CYP3A substrates, as observed in skin or other systems: 17β-estadiol, ethinylestradiol, progesterone, levonorgestrel, testosterone, dehydroepiandrosterone, cortisol, dexamethasone, acetaminophen, benzphetamine, nifedipine, midazolam, aflatoxin B_1_ (Oesch-Bartlomowicz and Oesch 2007).

##### CYP4 family

CYP4B1 mRNA was found in human skin (Baron et al. 2008), in human keratinocytes in culture (Saeki et al. 2002) and in the three-dimensional reconstructed human skin model EpiSkin^™^ (Luu-The et al. 2009).

Metabolism catalyzed by CYP4B1 includes the ω-hydroxylation of medium-chain fatty acids as well as the metabolic activation of several protoxins and pre-carcinogens. This includes the transformation of 4-ipomeanol to pneumotoxic metabolites and the transformation of many aromatic amines to reactive metabolites suggesting a potentially important role of CYP4B1 in toxicity and carcinogenesis for the skin.

### Non-CYP oxidoreductases

See also Tables 2, 11 and 12.

**Table 11:** Representative (more examples and references in the text) non-CYP-xenobiotic-metabolizing enzyme activities [constitutive; number after slash: induced (highest reported induced activity)] in human skin and human cells in culture

Substrate (preferentially for)	Skin	Keratinocytes^a^	HaCaT	NCTC 2544	KeratinoSens^®^	LuSens	U937	THP-1
Benzydamine (broad-spectrum FMO substrate)	+			bd	bd	bd	bd	
Arachidonic acid (COX)	23.5 ± 8.7^b^	5.1 ± 2.8^b^	0.05 ± 0.03^b^	0.02 ± 0.01^b^				
Ethanol (ADH)	0.3–0.4^c^				bd	bq	bd	bq
Propanal (ALDH)				8 ± 1.5^l^	4.77–16.4^c^	8.04–17.3^c^	bd	bq
2,6-Dichlorophenol indophenol (NQR)	~ 350^c^	~ 340^c,d^ ~ 880/1320^c,e^		134 ± 0.05^c^				
Menadione (NQR)	7–10^c^/~ 11^c,f^	~ 88^c^	~ 288^c^					
Fluorescein diacetate (E)	~ 0.5^g^	3.7^g^			3.20–4.06^g^	1.19–3.55^g^	0.911–1.057^g^	0.869–1.072^g^
4-MU heptanoate (E)		10–32^i^		23^i^				
CDNB (GST)	20–80^c^/290^c,h^	110–620^c^; ~ 50^i^	~ 47^i^	~ 40^i^ 167 ± 3^l^/234^l^				
1-Fluoro-2,4-dinitrobenzene (GST)			+					
1-Bromo-2,4-dinitrobenzene (GST)			+					
Para-nitrophenol (UGT)		0.016–0.023^i^						
4-MU (UGT)	1.3 ± 0.2^j^	bd to ~ 1.2^i^	1.99 ± 0.97^i^	~ 1^i^	bd	bd	bd	bd
PABA (NAT)	0.449 ± 0.175^g^	0.215 ± 0.070^g^		13.3–28.7^g^	10.9–22.6^g^	24.8–60.2^g^	6.00–29.9^g^	
		8 ± 0.5^k^	12.0–44.5^k^					
Para-toluidine (NAT)	0.63–3.03^c^	0.16 ± 0.08^i^	0.65 ± 0.37^i^	0.35 ± 0.22^i^				
2,4-Toluenediamine (NAT)	+	+						

*4-MU* 4-methylumbelliferone, *ADH* alcohol dehydrogenase, *ALDH* aldehyde dehydrogenase, *DCNB* 1-chloro-2,4-dinitrobenzene, *E* esterase, *FMO* flavin-dependent monooxygenase, *bd* below the limit of detection, *bq* below the limit of quantification, *NQR* NADH/NADPH quinone reductase, *PABA* para-aminobenzoic acid

^a^Keratinocytes = Primary keratinocytes in culture (“NHEC”, “Normal Human Epithelial Keratinocytes”)

^b^pg prostaglandin E2 formed/min/mg microsomal protein

^c^pmol/min/mg protein

^d^Differentiated keratinocytes

^e^Proliferating keratinocytes

^f^Ex vivo epidermis

^g^nmol/min/mg S9 protein

^h^Epidermis (scraped from skin surgical samples)

^i^nmol/min/mg protein, activity determined in culture medium

^j^nmol/min/mg microsomal protein

^k^nmol/min/mg cell lysate protein

^l^nmol/min/mg homogenate protein

**Table 12:** Representative (more examples and references in the text) non-CYP-xenobiotic-metabolizing enzyme activities [constitutive; number after slash: induced (highest reported induced activity)] in human skin and human reconstructed skin models

Substrate (preferentially for)	Skin	EpiDerm^™^	EpiDerm FT^™^	AST-2000	Episkin^™^	Episkin^™^ FTM	SkinEthik^™^ RHE	Phenion^®^ FT	StrataTest^®^	OS-REp	ORS-RHE
ORS-benzydamine (broad-spectrum FMO substrate)	+	5.95 ± 1.06^a^						4.02 ± 0.38^a^	0.5 ± 0.1^a^		
Arachidonic acid (COX)	23.5 ± 8.7^b^	3.6 ± 1.9^b^/~ 8^b^									
Ethanol (ADH)	0.3–0.4^c^	bd							< 36^c^		
Propanal (ALDH)									3.1 ± 0.8^c^		
2,6-Dichlorophenol indophenol (NQR)	~ 350^c^										
Menadione (NQR)	7–10^c^/~ 11^c,d^	~ 3.6^c^			~ 11^c^		~ 44^c^				
4-Methylumbelliferone acetate (E)	0.954 ± 179^e^				0.234 ± 0.049^e^	2.39 ± 0.13^e^	1.096 ± 0.257^e^				
Fluorescein diacetate (E)	~ 0.5^g^	0.8^g^	1.3^g^	0.59^g^				2.3 to ~ 5^g^	3.6 ± 0.1^g^		
Vitamin E acetate (E)	0.18^f^	0.12^f^									
CDNB (GST)	~ 290^c,h^	~ 410–920^c^			~ 180–430^c^		~ 390–850^c^	~ 40–90^c^		120–220^c^	
	15 ± 3^i^ (20 ± 6.8^c^)	(~ 62^c^)			51 ± 2^i^	112 ± 12^i^	30 ± 5^i^				
CDNB (microsomal GST)	~ 3^a^	~ 11^a^									
4-MU (UGT)	1.3 ± 0.2^a^	~ 1.8–1.98^a^						0.12 ± 0.05^a^	< 0.5^a^		
	18 ± 11^i^				68 ± 3^i^	54 ± 3^i^	8 ± 1^i^	~ 4–6^j^		~ 1–2.5^j^	
7-OH-Coumarin (UGT)											+
OH-BP^k^ (UGT)											+
OH-3-MC^k^ (UGT)											+
Para-nitrophenol (SULT) Traces					bd	bd	bd				
7-OH-Coumarin (SULT)											+
OH-BP^k^ (SULT)											+
OH-3-MC^k^ (SULT)											+
PABA (NAT)	33 ± 8^i^	11.2 ± 4.1^g^			21 ± 2^i^	47 ± 3^i^	60 ± 3^i^	17.0 ± 5.3^g^	7.2 ± 1.6^g^		
Para-toluidine (NAT)	0.63–3.03^c^	~ 0.68^c^									
2,4-Toluenediamine (NAT)	+		+					+			

For description of the reconstructed human skin models see Table 10

*4-MU* 4-methylumbelliferone, *bd* below detection, *CDNB* 1-chloro-2,4-dinitrobenzene, *E* esterase, *FMO* flavin-dependent monooxygenase, *NQR* NADH/NADPH quinone reductase, *OH-3-MC* hydroxylated 3-methylcholanthrene, *OH-BP* hydroxylated benzo[*a*]pyrene; *PABA* para-aminobenzoic acid

^a^nmol/min/mg microsomal protein

^b^pg prostaglandin E2 formed/min/mg microsomal protein

^c^nmol/min/mg cytosolic protein

^d^Ex vivo epidermis

^e^nmol/min/mg protein (sum of 7500 g supernatant + medium)

^f^nmol/h/cm^2^

^g^nmol/min/mg S9 protein

^h^Epidermis (scraped from skin surgical samples)

^i^pmol/min/mg protein (sum of 7500 g supernatant + medium)

^j^µmol/min/mg skin model protein

^k^Position of hydroxyl group unknown

#### Flavin-dependent monooxygenases (FMO)

A prominent reaction catalyzed by FMO is the N-oxygenation of many secondary and tertiary amines.

Further substrates include many other soft nucleophiles.

*FMO transcripts* Transcripts coding for all 5 FMOs (FMO 1–5) were discovered in human skin, FMO1, 2, 4 and 5 also in the three-dimensional human skin model EpiDerm^™^ (Hu et al. 2010). FMO transcripts are present in the human skin at levels greater to (or at least similar as) CYP transcripts (Janmohamed et al. 2001), yet most human FMO transcripts (namely those coding for FMO 1, 3, 4, and 5) were interindividually very variably expressed.

Flavin-dependent monooxygenase transcripts were reported to be localized in the human epidermis, sebaceous glands and hair follicles (Janmohamed et al. 2001), FMO1 predominantly in the epidermis, FMO2 and FMO3 predominantly in the dermis and FMO4 and FMO5 more ubiquitously in the total skin (Luu-The et al. 2009). Quite unexpectedly, the relative occurrence of FMO transcripts in three-dimensional human skin models did not mirror the one observed in the native skin. The level of FMO 1, 3, and 5 transcripts was extremely low in the epidermal model EpiSkin^™^. FMO2 transcript levels were high and FMO4 transcript levels were low in both, the epidermal and the full skin model (Luu-The et al. 2009). The differences in expression may be due to inhibitory or stimulatory factors present or absent in the native skin or in the culture medium, respectively.

Recently Wiegand et al. (2014) systematically and individually compared the expression of FMO mRNAs in several systems derived from the same three donors. FMO5 was expressed in all skin systems, with some preference to the epidermal systems. Thus, it was expressed highly in the epidermis of native skin and in the three-dimensional reconstructed skin epidermis model OS-REp, moderately in the epidermis of the full skin model Phenion FT Skin, moderately in the dermis of the native skin, weakly in the dermis of the full skin model Phenion FT Skin, weakly in the epidermal monolayer NHEK and in the monolayer fibroblasts, in each of these systems irrespective of which of the three donors they were originally derived. FMO3 mRNA, however, was, in contrast to the mRNAs coding for most of other xenobiotica-metabolizing enzymes, preferentially expressed in the dermis-derived systems. Thus, it was expressed weakly to moderately in the dermis of the native skin, weakly in the dermis of the full-thickness skin model Phenion FT Skin and in the monolayer fibroblasts, but it was expressed neither in the epidermis of the native skin nor in the epidermis of the full-thickness skin model Phenion FT Skin nor in the epidermal three-dimensional skin model OS-REp nor in the epidermal monolayer NHEK. However, all-*trans*-retinoic acid induced the expression of FM03 mRNA level after 24 h in the epidermis of the full-thickness skin model Phenion FT Skin and in the epidermal skin model OS-REp, where no constitutive expression of FMO3 mRNA was detected and led to an increase of the FMO3 mRNA expression in the dermal compartment of the Phenion FT Skin Model and in the fibroblast monolayers. FMO1 mRNA was consistently expressed (irrespective of which original donor) in native skin epidermis and dermis as well as in the three-dimensional skin models Phenion FT Skin and OS-REp, but only in two out of three donor-derived monolayer fibroblasts and in none of the NHEK monolayers.

Very recently Bacqueville et al. (2017) reported that of the FMOs only FMO4 mRNA was present in measureable amounts in their ORS-RHE model. It may be expected that FMO2 and FMO3 are lacking from epidermal models, because they are selectively expressed in the dermis (Luu-The et al. 2009; Wiegand et al. 2014). However, FMO1, FMO4 and FMO5 have been reported to be present in the epidermis or whole skin of native skin (Luu-The et al. 2009; Hu et al. 2010). Possible explanations for their lack in ORS-RHE may include effects of the culture medium (Luu-The et al. 2009) or the large variation of FMOs in skin from different donors (Oesch et al. 2014).

Janmohamed et al. (2001) reported that in the HaCaT cell line the levels of FMO 3 and 5 transcripts were similar to those in the native skin, but FMO4 transcript levels were considerably higher and FMO1 transcripts levels were below detection.

*FMO proteins* As shown by Western blot analysis FM03 was present at low levels in native human skin and was induced up to 20-fold after treatment with 10 µM all-*trans*-retinoic acid for 24 h (Wiegand et al. 2014). In NHEKs FMO3 protein, but no FMO1 protein was detected (Vyas et al. 2006).

With respect to *FMO enzymatic activities, cyclooxygenases, alcohol dehydrogenases*/*aldehyde dehydrogenases and NAD(P)H:quinone reductase in human skin and human skin models* (see Tables 7, 10, 11, 12) very little new information became available. For the status quo ante see our previous review Oesch et al. (2014). The only pieces of new information were as follows.

Very recently Bacqueville et al. (2017) reported that in their ORS-RHE model (Guiraud et al. 2014) *alcohol dehydrogenase 5* ((ADH)5) *mRNA* was expressed, while ADH3 [the most abundant isoform in skin (Cheung et al. 1999)] could not be probed, since it was not included in the gene panel used. ADH1B was absent consistent with its exclusively dermal location (Luu-The et al. 2009).

Bacqueville et al. (2017) very recently also reported that in their ORS-RHE model (Guiraud et al. 2014) the expression levels of 10 *aldehyde dehydrogenase* (ALDH) isoforms *mRNA* were good (Ct < 30) or strong (Ct < 25). ALDH3A1 was significantly induced (*p* < 0.001) by > twofold following the systemic application of beta-naphthoflavone.

In addition, Bacqueville et al. (2017) reported that in their ORS-RHE model (Guiraud et al. 2014) *NQR1 mRNA* was well expressed.

#### Aldehyde oxidase (AO)

AO activity has been described only recently in the skin (Manevski et al. 2014). Substrates of AO include azaheterocycles. To avoid CYP-mediated metabolism and also to reach a lower log *P* and to increase solubility azaheterocycles are often included into therapeutics. AO also oxidizes endogenous and exogenous aldehydes to carboxylic acids (Manevski et al. 2014). Missing information on the action of AO had led to failures in drug development (Kaye et al. 1984; Dittrich et al. 2002; Dalvie et al. 2010; Diamond et al. 2010; Akabane et al. 2011; Zhang et al. 2011). Cutaneous AO gene expression at the level of mRNA formation (Hu et al. 2010) and protein formation (van Eijl et al. 2012) had semiquantitatively been described, but AO activity and substrate specificity has been reported only very recently (Manevski et al. 2014). Enzyme activities toward the specific AO substrates carbazeran and zoniporide tested in healthy full-thickness skin from 13 human donors showed remarkable reaction rates comparable to those of cutaneous glucuronidation, sulfation, and N-acetylation. Carbazeran was metabolized to 4-hydroxycarbazeran and zoniporide to 2-oxo-zoniporide with average rates of 1.30 and 0.164 pmol/h/mg skin leading to 13 and 2% substrate turnover, respectively, after 24 h incubation of 10 mM substrate. Interindividual variabilities were threefold for zoniporide and sixfold for carbazeran (Manevski et al. 2014).

#### Other oxidoreductases

Very recently Bacqueville et al. (2017) reported that in their ORS-RHE human skin model (Guiraud et al. 2014) *hydroxysteroid 17-β dehydrogenase 10* (HSD17B10) was strongly expressed. This enzyme is resident in the basement layer of the epidermis and is involved in estrogen formation and metabolism (Hikima and Maibach 2007).

*Skin surface bacteria* have also been shown to be able to reductively metabolize xenobiotic compounds, most notably converting azo dyes to aromatic amines (Korinth et al. 2013). Bacterial reduction of the azo dye Direct Blue 14 led to the formation of o-toluidine. Most bacterial strains of normal human skin flora exhibit reductive activity. Thus, in a synthetic sweat suspension they cleaved Direct Blue 14. The reductive cleavage of azo dyes by several skin surface bacteria achieved 100% within less than 5 h as demonstrated on Methyl Red and Orange II (Korinth et al. 2013 and references quoted therein). Thus, as discussed above, it should be kept in mind that the microbiome on the skin and its potentially quite divergent abundance and composition in vivo versus in vitro may substantially contribute to differences of results of in vivo experiments versus in vitro models, possibly even versus ex vivo experiments.

With respect to *Hydrolases and Conjugating enzymes in human skin and human skin models* (see Tables 7, 10, 11, 12) very little new information became available. For the status quo ante see our previous review Oesch et al. (2014). The only pieces of new information were as follows:

Very recently Bacqueville et al. (2017) reported that in their ORS-RHE model (Guiraud et al. 2014) the preferentially in the dermis expressed *microsomal epoxide hydrolase (EPHX1) transcripts* were expressed at low up to good levels.

With respect to recent findings concerning *esterases* Bacqueville et al. (2017) reported that in their ORS-RHE model (Guiraud et al. 2014) UCHL3 (ubiquitin carboxyl-terminal esterase L3, a protease) and ESD (esterase D, involved in the recycling of sialic acids) mRNAs were strongly expressed, while carboxylesterase 1 and 2 (CES1/2) which play an important role in the hydrolysis of a large number of structurally diverse drugs (Imai 2006; Oesch et al. 2007) were expressed at good levels in ORS-RHE. CES3 which is not expressed in native human skin (Hu et al. 2010), was not expressed in ORS-RHE either (Bacqueville et al. 2017).

Hopf et al. (2014) reported recently that the softening plasticizer and suspected endocrine disruptor diethyl hexyl phthalate is completely hydrolysed to monoethyl hexyl phthalate by freshly excised human skin such that only this metabolite and an oxidative metabolite, 5-oxo-monoethy hexyl phthalate, derived from the primary metabolite penetrate through the skin into the receptor fluid.

With respect to recent findings concerning *glutathione S-transferases* (GST) Wiegand et al. (2014) systematically and individually compared the *expression* of GSTP1 mRNAs in several systems derived from the same three donors. GSTP1 mRNA was expressed in all skin systems, with some preference to the epidermal systems. Thus, it was expressed moderately (1 of 3 donors) to highly (2 of 3 donors) in the epidermis of native skin and weakly (1/3 donors) to moderately (2/3 donors) in the dermis. It was moderately expressed in the human epidermis-derived NHEK (3/3 donors) and in the human fibroblast (3/3 donors) monolayer cultures. It was moderately (1/3 donors) to strongly (2/3 donors) expressed in the human epidermis-derived three-dimensional skin model OS-REp, moderately (1/3 donors) to strongly (2/3 donors) in the epidermis of the full-thickness skin model Phenion FT Skin and moderately (3/3 donors) in the dermis of this model. Very recently Bacqueville et al. (2017) reported strong expression of GSTP1 in their ORS-RHE model (Guiraud et al. 2014).

Wiegand et al. (2014) observed a very similar *GST activity* toward CDNB (80 nmol/min/mg protein) in human full skin biopsies, at somewhat higher levels in the epidermis (100 nmol/min/mg protein) compared with the dermis (40 nmol/min/mg protein). Melanocytes in the basal layer of the epidermis possess significant GST activity mainly due to GSTP1 (Zhang et al. 2002). Jacquoilleot et al. (2015) reported recently GST activity toward different halo-dinitrobenzenes in the human epidermal cell line HaCaT by measuring GSH depletion. They observed that after rapid depletion (within the first h of exposure), GSH was repleted to levels similar to untreated control cells within 24 h after exposure to CDNB and 1-fluoro-2,4-dinitrobenzene, but not after exposure to 1-bromo-2,4-dinitrobenzene. In line with this, 1-bromo-2,4-dinitrobenzene was the most toxic of the three halo-dinitrobenzenes in all assays. However, all three halo-dinitrobenzenes at concentrations of 5–10 µM activated the Nrf2 pathway. Recently Wiegand et al. (2014) reported that GST activities toward CDNB were higher in monolayers of the epithelial cell-derived NHEK (around 110 nmol/min/mg protein, estimated from the figure) than in the fibroblasts (around 40 nmol/min/mg protein) similar to the epidermal versus dermal compartment of the native skin (see above). Recently Wiegand et al. (2014) observed in the epidermal compartment of the full-thickness human skin model Phenion FT higher GST activity toward CDNB (around 90 nmol/min/mg cytosolic protein, estimated from the figure) then in the dermal compartment (around 40 nmol/min/mg cytosolic protein) which is similar to these layers in the native skin discussed above. The highest activity (120–220 nmol/min/mg protein) in their comparative study was observed in the epidermal three-dimensional model OS-REp.

With respect to recent findings concerning *UGT-glucuronosyltransferases* Wiegand et al. (2014) systematically and individually compared the *expression* of UGT1A10 mRNAs in several systems derived from the same three donors. UGT1A10 mRNA was expressed in almost all skin systems, with some preference to the epidermal systems. Thus, it was expressed highly in the epidermis of native skin and moderately in the dermis of all three donors. It was moderately expressed in the human epidermis-derived NHEK monolayer cultures, but in none of the human fibroblasts. It was strongly expressed in the human epidermis-derived three-dimensional skin model OS-REp, moderately in the epidermis of the full-thickness skin model Phenion FT Skin and weakly to moderately in the dermis of this model. Very recently Bacqueville et al. (2017) reported that several UGT family 1A mRNAs, most notably UGT1A10, were expressed in their ORS-RHE model (Guiraud et al. 2014). The expression of UGT1A3/5/6/7/8 and 10 mRNAs were induced by 3-MC and beta-naphthoflavone two- to fivefold.

Concerning recent findings on *UDP-glucuronosyltransferase enzymatic activities* Manevski et al. (2015) reported glucuronidation for human skin (fresh whole skin explants) for the first time for the following substrates: indomethacin, diclofenac and 17b-estradiol. From the former two substrates acyl glucuronides were formed, from the latter substrate 3- and 17-glucuronides were formed. Interindividual variability was relatively high (13.0-fold) for 4-methylumbelliferone and diclofenac glucuronidation (possibly due to an eyelid skin sample from a male individual which glucuronidated 4-methylumbelliferone and diclofenac approximately threefold faster than the average). Freezing of the fresh skin led to loss of glucuronidation activity. Wiegand et al. (2014) reported similar UGT activities toward 4-methylumbelliferone of about 1–2 nmol/min/mg protein in NHEK and in human fibroblasts. These authors (Wiegand et al. 2014) reported rather high UGT activities toward 4-methylumbelliferone (4–6 µmol/min/mg protein) in the full-thickness reconstructed human skin model Phenion FT with similar activities in its epidermal and dermal compartment. The activities were also quite high, but somewhat lower (about 1–2.5 µmol/min/mg protein) in the epidermal model OS-REp. Very recently Bacqueville et al. (2017) reported that in their ORS-RHE model 7-ethoxycoumarin was via 7-hydroxycoumarin efficiently metabolized to the 7-glucuronide, presumably by UGT 1A6 (Lampe et al. 1999), which they also had found to be well expressed in their model. BP and 3MC were metabolized via their hydroxylated metabolites to glucuronides.

With respect to recent findings on *sulfotransferase* regulation of SULT2B1b was observed at the *transcriptional level*. SULT2B1b mRNA (as well as protein and enzyme activity) increased with calcium-induced differentiation in keratinocytes. SULT2B1b expression is increased by LXR and PPAR activation in human keratinocytes. SULT1E1 also is expressed in keratinocytes. It also is substantially upregulated during keratinocyte differentiation which minimizes the pro-proliferative effect of estrogen (Runge-Morris et al. 2013 and references therein).

- Very recently Bacqueville et al. (2017) reported that SULT2B1, SULT1A3, SULT1E1 (and thiosulfate sulfurtransferase, also called rhodanese) mRNAs were expressed in good levels in their ORS-RHE model (Guiraud et al. 2014).

With respect to recent findings on *sulfotransferase enzymatic activity* (Sharma et al. 2013a, b) showed that human (and rat) skin SULT is responsible for biotransformation of the major nevirapine (Viramune) metabolite 12-hydroxynevirapine to the corresponding reactive benzylic sulfate and that this, in turn, leads to covalent binding to proteins (Sharma et al. 2013a) and to the severe immune-mediated skin rash (Sharma et al. 2013b) caused (in humans and in rats) as a serious side effect of the HIV combatting drug nevirapine (none of this occurs in the mouse skin: no 12-sulfate, no covalent binding, no skin rash; thus, although the mouse is widely used for immunological studies, for this metabolism-dependent immunotoxicity the rat, but not the mouse, appears to be a suitable model for human). Beside of leading to covalent binding to proteins, the SULT-generated nevirapine 12-sulfate led to the upregulation of a number of genes potentially also involved in toxic side effects of nevirapine. Markedly (3- to 18-fold) upregulated genes include S100A7 considered a danger signal, IL-22 RA2 marking an immune response, TRIM63, a ubiquin ligase and TRIM proteins which play a role in inflammasome activation, DAPK1 participating in inflammasome assembly through binding to NLRP3, an NOD-like receptor expressed in keratinocytes, all of this potentially contributing to the induction of nevirapine-induced skin rash (Zhang et al. 2013). In vitro incubation of the primary nevirapine metabolite 12-hydroxynevirapine with the SULT cofactor PAPS and recombinant SULT1A1*1, a human isoform that is present in the skin, also led to covalent binding, which was inhibited by the SULT inhibitor1-phenyl-1-hexanol implying that this SULT isoform was involved in the toxication of nevirapine (Sharma et al. 2013b). SULT2B1b (which is expressed in tissues that do not express SULT2A1, including skin) biotransforms 3β-hydroxysteroids such as pregnenolone and dehydroepiandrosterone to their sulfates (Runge-Morris et al. 2013 and references therein). Especially important for the skin, it converts (with high affinity) cholesterol to the amphipathic sulfolipid cholesterol sulfate. It is expressed in differentiating human keratinocytes. There it generates high levels of cholesterol sulfate which is important for dermal lipid homeostasis and which plays a major role in regulating several keratinocyte functions such as corneocyte desquamation. Cholesterol sulfate functions as a signaling molecule that promotes keratinocyte differentiation through activation of the protein kinase C isoform PKCη. As keratinocytes move upward through differentiating layers of epithelium, they produce increasing amounts of cholesterol sulfate. Most recently Bacqueville et al. (2017) reported that in their three-dimensional model ORS-RHE 7-ethoxycoumarin was via 7-hydroxycoumarin metabolized to the 7-sulfate possibly by any or all of the SULT isoforms which they had shown to be expressed in their ORS-RHE model (see above). BP and 3MC were metabolized via their hydroxylated metabolites to sulfates.

With respect to recent findings on *N-acetyltransferase transcript expression* Wiegand et al. (2014) systematically and individually compared the expression of NAT1 mRNAs in several systems derived from the same three donors. NAT1 was expressed in all skin systems, with some preference to the epidermal systems. Thus, it was expressed highly in the epidermis of native skin and moderately in the dermis of all three donors. NAT1 mRNA was expressed in the human epidermis-derived NHEK (moderately in those derived from two donors, weakly in those derived from one donor) and in the human fibroblasts (weakly in those derived from any of the three donors). In the recent systematic and individual comparison by Wiegand et al. (2014) NAT1 mRNA was expressed in the human epidermis-derived three-dimensional skin model OS-REp (strongly in that derived from one donor, moderately in those derived from the other two donors) and weakly (two donors) to moderately (one donor) in the epidermis of the full-thickness skin model Phenion FT Skin and weakly (all three donors) in the dermis of this full-thickness model. Very recently Bacqueville et al. (2017) reported that NAT1, but not NAT2 mRNA was expressed in their ORS-RHE model (Guiraud et al. 2014).

With respect to *N-acetyltransferase enzymatic activity* recently Manwaring et al. (2015) reported N-acetylation as the major pathway of metabolism in human skin ex vivo for several aromatic amine hair dyes. Thus, after 60 min exposure to 1.5 mg/cm^2^ skin in receptor fluid collected for 24 h the percentage of acetylated metabolite was 86% for 4-amino-2-hydroxytoluene and 72% for 4-amino-*m*-cresol, after 60 min exposure to 1.0 mg/cm^2^ skin 85% for 2-amino-5-ethylphenol, after 60 min exposure to 2.22 mg/cm^2^ skin 90% for toluene-2,5-diamine. Recently Manevski et al. (2015) reported for human skin (fresh whole skin explants) N-acetylation for the first time for procainamide as substrate. Of totally 11 substrates for five xenobiotic-metabolizing enzyme activities (glucuronidation, sulfation, N-acetylation, catechol methylation, and glutathione conjugation) the highest observed activity in human skin (fresh whole skin explants) was N-acetylation of para-toluidine (4.76 pmol/mg skin/h) (Manevski et al. 2015). Recently Manwaring et al. (2015) reported N-acetylation as the major pathway of metabolism in the human keratinocytic cell line HaCaT for several aromatic amine hair dyes. Thus, after 24 h incubation of 0.625 µg/mL in the medium the percentage of acetylated metabolite was 79% for 4-amino-2-hydroxytoluene, after 24 h incubation of 0.492 µg/mL 100% for 4-amino-*m*-cresol, after 24 h incubation of 0.5 µg/mL 100% for 2-amino-5-ethylphenol, after 24 h incubation of 0.35 µg/mL 100% for toluene-2,5-diamine and after 24 h incubation of 0.59 µg/mL 100% for *p*-phenylenediamine. In HaCaT cells Zeller and Pfuhler (2014) demonstrated the monoacetylated and the diacetylated metabolites had completely lost the genotoxic activity of their precursor aromatic amines *p*-phenylenediamine and 2,5-diaminotoluene and so did the *N*-acetylated metabolite of 4-amino-2-hydroxytoluene. For the bifunctional aromatic amines studied (*p*-phenylenediamine and 2,5-diaminotoluene), monoacetylation was sufficient to completely abolish their genotoxic potential. Similar results were obtained in the *Salmonella typhimurium* reversion assay, micronucleus test with cultured human lymphocytes (using 4-amino-2-hydroxytoluene), chromosome aberration assay with V79 cells (using 2,5-diaminotoluene) and Comet assay performed with V79 cells. Detoxication through N-acetylation was confirmed by comparing *p*-phenylenediamine, 2,5-diaminotoluene and 4-amino-2-hydroxytoluene in the Comet assay using the NAT-proficient HaCaT cells and V79NAT1*4 cells which specifically express the human NAT1 enzyme NAT1*4 with standard V79 cells (NAT deficient). In the NAT-proficient cells they observed dose–response curves which were clearly shifted to higher concentrations of these aromatic amines with no significant (*p* < 0.05) genotoxicity at the lowest concentrations tested. From these results the authors imply that NAT1 in the human skin may function as a first-pass elimination and only doses of (the tested) aromatic amines which overwhelm the human skin NAT1 may exert genotoxicity (Zeller and Pfuhler 2014). Most recently Grohmann et al. (2017) observed the mono *N*-acetylated derivative *N*-(3-amino-4-methylphenyl)acetamide as the only metabolite formed in substantial amounts from the carcinogenic 2,4-toluenediamine in two commercially available reconstructed human skin models (RHS Phenion^®^FT and EpiDermTMFT), human skin ex vivo, primary epidermal keratinocytes, dermal fibroblasts, epidermal Langerhans cells and dermal dendritic cells, which indicates the predominance of N-acetylation of 2,4-toluenediamine and, by inference, in all likelihood quite generally of aromatic amines, over other metabolic conversions in nice agreement in all these systems including human skin ex vivo.

With respect to recent findings on *other cutaneous acyltransferases* Bacqueville et al. (2017) reported that SAT1 (spermine *N*1-acetyltransferase 1, involved in the regulation of the intracellular concentration of polyamines) and NAA20 (*N*[alpha]-acetyltransferase 20) mRNAs were strongly expressed in their ORS-RHE model (Guiraud et al. 2014).

*Catechol O-methyltransferase (COMT)* Very recently Manevski et al. (2015) reported for human skin (fresh whole skin explants) for the first time methoxy derivatives as metabolites of 4-nitrocatechol and 2,3-dihydroxynaphthalene, which were formed at a rate of 1.15 and 1.78 pmol/h/mg skin (sum of extraction of explant and of medium), respectively. In line with this, very recently Bacqueville et al. (2017) reported that COMT mRNA was strongly expressed in their ORS-RHE model (Guiraud et al. 2014).

## Conclusions

### Attempt of recommendations derived from the collected information

As discussed above, important new information has become available since our last review (Oesch et al. 2014), but the recommendations derived from the hitherto available information remain largely similar.

#### Attempt of recommendation of models for xenobiotic-metabolism-dependent dermal absorption

Dermal absorption is largely dependent on the molecular size and on the relative lipophilicity of the compound under consideration. Hydrolases decrease the molecular size and conjugases increase the molecular size, while oxido reductases often substantially change the lipophilicity of the compounds in question. Esterases are especially important for dermal absorption, since they convert larger molecules into smaller ones and, in addition, convert lipophilic esters into more hydrophilic products.

With respect to animal models for the human skin, pig skin esterase activities are close to those of the human skin as far as information is available. With respect to in vitro models, the esterase activities of a considerable number of human models are reasonably close (within one order of magnitude) to native human skin. These include: primary human keratinocytes and the following cell lines: NCTC 2544, LuSens, KeratinoSens^®^, U937, THP-1 as well as the following three-dimensional human skin models: AST-2000, EpiDerm^™^, SkinEthik^™^ and StrataTest^®^—AST-2000 and SkinEthik^™^ almost identical. For estimating the relative importance of further xenobiotica-metabolizing enzymes see Table 13 to aid choosing the most appropriate model for a compound in question. Quite generally, it also has to be considered that the presently available in vitro skin absorption models do not possess a barrier mimicking the barrier of the native skin.

**Table 13:** Attempt to approximate an estimation of relative suitability of human skin models with respect to various xenobiotic-metabolizing enzymes. Reproduced from Oesch et al. (2014)

Enzyme	Presumably suitable models^a^
CYP 1 family^b^	*Primary human keratinocytes*, HaCaT, NCTC 2544, *Episkin*^*™*^, *Episkin*^*™*^ *FTM, SkinEthik*^*™*^ *RHE*
CYP2B6^c^	*Primary human keratinocytes, NCTC 2544*
CYP3A^d^	Primary human keratinocytes, *HaCaT, NCTC 2544, EpiDerm*^*™*^
COX^e^	Primary human keratinocytes, EpiDerm^™^
NQR	*Primary human keratinocytes*^*f*^, *NCTC 2544*^f^, *EpiDerm*^*™* g^, *Episkin*^*™* g^, SkinEthik^™^ RHE^g^
Esterase^h^	Primary human keratinocytes, *NCTC 2544*, KeratinoSens^®^, LuSens, *U937, THP-1, EpiDerm*^*™*^, *EpiDermFT*^*™*^, *AST-2000*, Episkin^™^, *Episkin*^*™*^*FTM, SkinEthik*^*™*^*RHE*, Phenion^®^ FT, StrataTest^®^
Cytosolic GST^i^	*Primary human keratinocytes, HaCaT*, N*CTC 2544, EpiDerm*^*™*^, *Episkin*^*™*^, Episkin^™^ FTM, *SkinEthik*^*™*^ RHE
Microsomal GST^i^	EpiDerm^™^
UGT^i^	*EpiDerm*^*™*^, Episkin^™^, *Episkin*^*™*^ *FTM, SkinEthik*^*™*^ *RHE*
*NAT*	*Primary human keratinocytes*^k^, *HaCaT*^k^, *NCTC 2544, EpiDerm*^*™* l^, *Episkin*^*™* k^, *Episkin*^*™*^ *FTM* ^k^, *SkinEthik*^*™*^ *RHE* ^k^, *Phenion*^®^ *FT*^m^, StrataTest^® m^

*4-MU* 4-methylumbelliferone, *BQOD* benzyloxyquinoline *O*-dealkylase, *CDNB* 1-chloro-2,4-dinitrobenzene, *COX* cyclooxygenase, *CYP* cytochrome P450, *ECOD* 7-ethoxycoumarin-*O*-dealkylase, *EROD* 7-ethoxyresorufin-*O*-deethylase, *GST* glutathione *S*-transferase, *MROD* 7-methoxyresorufin-*O*-demethylase, *NQR* NADH/NADPH quinone reductase, *PABA* para-aminobenzoic acid, *PROD* pentoxyresorufin *O*-depentylase, *NAT N*-acetyltransferase, *UGT* UDP-glucuronosyltransferase

^a^According to the present state of information. To be taken with great caution because of very limited comparability of data in most cases (see data and footnotes in Tables 8, 9, 11, 12 and text). Selected for this table if activities in model compared with human skin appear to be within one order of magnitude, underlined if within a factor of 3

^b^Estimated using EROD activities. Not applicable for CYP1A2 (based on MROD activities). Reliability of numbers very limited because of technical difficulties of accurate determination of very low CYP activities

^c^Estimated using PROD activities. Reliability of numbers very limited because of technical difficulties of accurate determination of very low CYP activities

^d^Estimated using BQOD and erythromycin *N*-demethylase activities. Reliability of numbers very limited because of technical difficulties of accurate determination of very low CYP activities

^e^Estimated using arachidonic acid as substrate

^f^Estimated using 2,6-dichloroindophenol as substrate

^g^Estimated using menadione as substrate

^h^Estimated using 4-methylumbelliferone acetate or fluorescein diacetate as substrate

^i^Estimated using CDNB as substrate

^j^Estimated using 4-MU as substrate

^k^Estimated using PABA as substrate

^l^Estimated using para-toluidine as substrate

^m^Indirect comparison: Phenion^®^ FT and StrataTest^®^ compared to EpiDerm^™^ with para-aminobenzoic acid as substrate; EpiDerm^™^ compared to human skin with para-toluidine as substrate

#### Attempt of recommendation of models for xenobiotic-metabolism-dependent genotoxicity and sensitization

The target molecules mediating *genotoxicity and sensitization* are different: for genotoxicity DNA, for sensitization proteins. Yet in most instances genotoxicity and sensitisation are both caused by electophilically reactive species and the enzymes generating or removing these reactive metabolites are largely the same. An intrinsic problem for respective generalizations and predictions is that so far all adequately investigated XME which are toxifying some substrates have been found to detoxify other substrates [to the best of our knowledge we were the first to establish and point this out for a XME by demonstrating the dual role of microsomal epoxide hydrolase in both, activation and inactivation (Bentley et al. 1977)].

Despite of the fact that CYPs also play dual roles in—depending on the compound in question—sometimes mediating toxication and sometimes detoxication, their predominant role is toxication. The opposite is true for hydrolytic and conjugating enzymes: depending on the compound in question they sometimes mediate detoxication and sometimes toxication, but predominantly they mediate detoxication. To approximate the relative suitability of models for the native human skin it may be considered which enzymes act predominantly in one of these two directions, preferentially with respect to the individual compound in question.

Among *animal models* cutaneous *CYPs* are relatively close to human skin in rats and pig although the paucicity of available data does not allow a strong conclusion. On the other hand, CYP activities in the mouse skin are generally much higher than those in the human skin. As a striking example for better predictions if the individual compound is taken in focus: quite to the contrary of the situation with respect to CYPs just discussed before, levels of *NADH*/*NADPH quinone reductase* (NQR) are quite similar in mouse skin compared with the human skin and with respect to *microsomal epoxide hydrolase* (EPX1) the mouse skin appears to be at least a considerably better model for the human skin than the rat skin. *Glutathione S-transferase* (GST) levels in both, the mouse skin and the rat skin are quite similar to human skin (except GSTA4-4). For *UGT-glucuronosyltransferase, sulfotransferase* and *N-acetyltransferase* not sufficient comparable data are available for a meaningful estimation of the relative suitability of the individual animal models with respect to their activities in the human skin.

Taken together, a judgement on the relative goodness for predictions will largely depend on whether CYPs, NQO, hydrolases or GST may be limiting for the control of electrophilically reactive species. However, if the last-mentioned enzymes (UGT, SULT, NAT) are expected to also play a relevant role for the control of electrophilically reactive species, even an only approximate judgment appears at present not possible. A single exception from this may be the group of compounds with structural elements allowing for the metabolic generation of reactive benzylic sulfates. For this group of compounds the recent results reported by Sharma et al. (2013a, b) indicate that the rat skin is a much better model for the human skin than the mouse skin, but from the information available so far it is still not possible to estimate the limits for generalizations.

The relative suitability of *human in vitro models* with respect to *CYP* activities is hampered (a) by the very low activities of most individual CYPs in most of these models and (b) by the fact that individual models which appear quite reasonably close to the native human skin with respect to a given substrate in several instances turn out to be especially bad with respect to other substrates. This reiterates the important point, that predictions will become better when individualized to the compound in question and to the individual CYP(s) which predominantly metabolize(s) the substrate in question. Thus, judgment which human skin in vitro model with respect to reactive-metabolite-producing CYPs is closest or sufficiently close to the native human skin largely depends on the structure of the compound in question which limits the possibilities for generalizations and the very low (to not detectable) CYP activities constitute a further serious limitation.

The situation is somewhat better with respect to *COX* in that its specific activities with arachidonic acid as substrate are easily measurable. In primary keratinocytes and in EpiDerm^™^ COX activities are reasonably close to the human skin (within one order of magnitude), while Cox activities in NCTC 2544 and HaCat cell lines are very different from those in the native human skin (500- to 1000-fold different).

For the *NADH*/*NADPH quinone reductase* (NQR) the specific activities appear in primary human keratinocytes, in the cell line NCTC2544, in the reconstructed human skin models EpiDerm^™^, Episkin^™^ and SkinEthik^™^ within an order of magnitude compared with human skin, in EpiDerm^™^ and Episkin^™^ very close to human skin.

*Glutathione S-transferase* activities for 1-chloro-2,4-dinitrobenzene (CDNB) are reasonably close (within one order of magnitude) compared with native human skin in the following human in vitro cells/cell lines: primary human keratinocytes, HaCaT and NCTC 2544. In the three-dimensional skin models Episkin^™^ FTM, EpiDerm^™^, Episkin^™^, SkinEthik^™^ RHE and OS-Rep GST activities suitable for comparisons are reasonably close to human skin, in the latter four models in average even within a factor of about 3.

*UGT-glucuronosyltransferase* activities toward 4-methylumbelliferone in NCTC2544 and HaCaT cell lines cannot be compared with human skin, but are at least close (within a factor of 2) to those in primary human keratinocytes. The activities toward this substrate are within a factor of 5 in most of the 3-dimensional human skin models (EpiDerm^™^, Episkin^™^, Episkin^™^ FTM and SkinEthik^™^ RHE), within a factor of about 10 in Phenion^®^FT.

The *N-acetyltransferase activities* suitable for comparisons are relatively close (directly or indirectly compared within a factor of about 3) to those in human skin (within a factor of 3) in the following human in vitro models: primary human keratinocytes, HaCaT cell line and in the three-dimensional models EpiDerm^™^, Episkin^™^, Episkin^™^ FTM, Phenion^®^ FT, SkinEthik^™^ and StrataTest^®^.

In conclusion, predictions on the relative suitability of in vitro human models for the human skin to predict genotoxicity and sensitization are very limited for CYPs. However, for phase II enzymes the following human in vitro models may in full awareness that the information available is still scarce—be cautiously taken as presumably reasonably adequate: primary human keratinocytes and the three-dimensional human skin models, especially Epiderm^™^, Episkin^™^, Episkin^™^ FTM and SkinEthik^™^ RHE. If it is possible to estimate from the structure of the compound in question which enzymes may be most important/most limiting in the control of the reactive metabolite(s) responsible for the genotoxic and/or sensitizing effects in question, Table 13 may aid in deciding which model may be best suited for predictions.

#### Attempt of recommendation of models for xenobiotic-metabolism-dependent skin irritation

For predicting potential skin irritation cutaneous metabolism is important when generating or detoxifying skin irritating species. Esterases often convert non-irritant esters into irritant alcohols which are further metabolized to aldehydes and these to carboxylic acids. Thus, consideration of cutaneous metabolism is especially important for attempting correct predictions of skin irritation in human skin by esters, implying that the model should preferably be close to human skin with respect to esterases, alcohol dehydrogenases and aldehyde dehydrogenases.

Among *animal models*, the pig appears to be a good model for *esterase* activities although the limitation must be kept in mind that not much is known about substrate specificities. Pig skin appears to be a considerably better model than the rat skin, while on the skin of mouse and guinea pig clearly insufficient information is available. Rat, mouse and guinea pig skin cutaneous *alcohol dehydrogenase* activities appear to be reasonably close (within one order of magnitude) to those in human skin.

Among human *in vitro models esterase* activities with fluorescein diacetate as substrate are relatively close (within a factor of 8) compared with human skin S9 in the cell lines KeratinoSens^®^, LuSens, THP-1 and U937 (in the latter two very close). Esterase activities are reasonably close (within one order of magnitude) compared with native human skin in all three-dimensional skin models summarized in this review, almost identical in AST-2000 with fluorescein diacetate as substrate and in SkinEthik^™^ RHE with 4-methylumbelliferone acetate as substrate. In EpiDerm^™^ esterase activity with vitamin E as substrate was very close to the activity found in human skin.

If for a given compound it can be estimated which cutaneous enzyme(s) are crucial in the control of the potential skin irritating properties Table 13 may be helpful for deciding which model may be best predictive.
