# Correction to: Xenobiotica-metabolizing enzymes in the skin of rat, mouse, pig, guinea pig, man, and in human skin models

**DOI:** 10.1007/s00204-018-2301-1

**Published:** 2018-09-12

**Authors:** F. Oesch, E. Fabian, Robert Landsiedel

**Affiliations:** 1 Institute of Toxicology, Johannes Gutenberg-University, Obere Zahlbacherstr. 67, 55131 Mainz, Germany; 2 Experimental Toxicology and Ecology, GV/TB, Z470, BASF SE, Carl-Bosch-Str. 38, 67056 Ludwigshafen, Germany

## Correction to: Archives of Toxicology (2018) 92:2411–2456 10.1007/s00204-018-2232-x

This article is published with the incorrect copyright holder name in the HTML article as “© Springer 2018”. The correct copyright line should read “The Author(s) 2018” (as it appears in the article PDF).

